# Quantum Zeno Effect Permits Magnetosensitivity in Lipid Peroxidation despite Fluctuating Inter-Radical Coupling

**DOI:** 10.1021/jacsau.6c00031

**Published:** 2026-04-15

**Authors:** Matt C. J. Denton, Daniel R. Kattnig

**Affiliations:** † Living Systems Institute, 3286 University of Exeter, Stocker Road, Exeter, Devon EX4 4QD, U.K.; ‡ Department of Physics, University of Exeter, Stocker Road, Exeter, Devon EX4 4QL, U.K.

**Keywords:** magnetic field effects, radical pair mechanism, lipid peroxidation, Brownian motion, quantum Zeno effect

## Abstract

Lipid peroxidation, the oxidative degradation of lipids, occurs via a complex radical chain reaction and is central to biological processes such as cellular aging and ferroptosis. It is also linked to numerous pathologies, often marked by excessive oxidative stress. Growing evidence implies that lipid peroxidation is sensitive to weak magnetic fields, with the radical pair mechanism (RPM) proposed as a possible explanation. Previous studies have demonstrated these effects with simple models but have yet to evaluate the efficacy of the RPM under biologically realistic conditions. For a complete picture, models must include the effects of strong inter-radical interactions, which typically suppress magnetic field sensitivity and expedite spin relaxation. Using Brownian dynamics-informed spin dynamics calculations, we investigate the impact of dynamic inter-radical dipolar coupling on the predicted magnetic field sensitivity of the chain termination reaction. We find that weak magnetic field effects persist despite strong, fluctuating dipolar interactions, provided that the spin-selective radical recombination is sufficiently fast to induce the quantum Zeno effect. Under such conditions, the recombination quantum yield exhibits a strong dependence on the recombination rate constant, with certain rates giving rise to low-field effects, while others enhance high-field sensitivity or eliminate the magnetic responsiveness altogether. At high magnetic fields, spin relaxation driven by *g*-matrix anisotropy dominates, potentially leading to pronounced magnetosensitivity for fast recombination processes. Overall, our results demonstrate that magnetic field effects are viable in strongly coupled radical pairs within biological membranes, given appropriate dynamical and kinetic constraints, and highlight the potential for broader magnetosensitivity in confined, low-mobility biological environments than previously anticipated based on standard RPM models.

## Graphical abstract

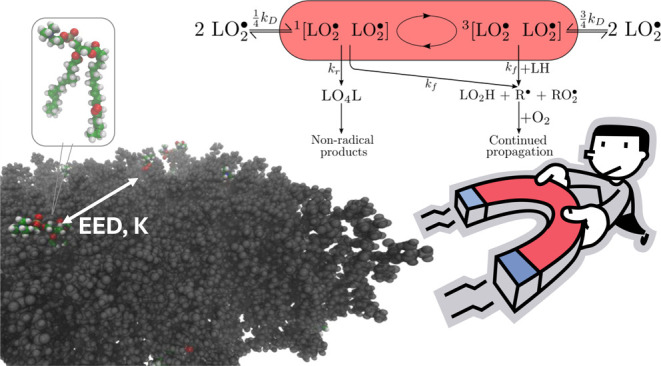

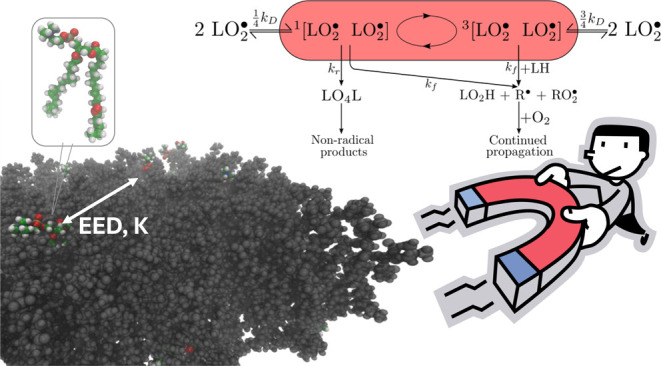

## Introduction

Lipid peroxidation is the name given to the oxidative degradation of lipids and is a ubiquitous process in biological systems.^1^ Elevated levels of lipid peroxidation are closely associated with oxidative stress, cellular aging, and various diseases.^2–4^ Recent experimental studies suggest that this process may be sensitive to weak magnetic fields,^5–9^ and theoretical investigations propose that the radical pair mechanism (RPM) acting on the termination step of the underlying chain reaction is a possible explanation for this.^10,11^

Lipid peroxidation proceeds through a radical chain reaction in which lipid peroxyl radicals (LO_2_^•^), embedded within lipid membranes, serve as key chain carriers.^12^ These radicals originate from the reaction between lipid molecules (LH) and reactive oxygen species (ROS), typically by the abstraction of hydrogen atoms from unsaturated lipid tails.^13^ The resulting lipid radicals (L^•^) incorporate molecular oxygen to form lipid peroxyl radicals (LO_2_^•^), which are the predominant radical species in membranes.^10,14^ These reactions comprise the initiation phase of the chain process (Figure 1A). In the propagation phase, LO_2_^•^ abstracts hydrogen atoms from adjacent lipid molecules, generating a L^•^, which again integrates oxygen, thereby effectively producing an oxidized—that is, damaged—lipid hydroperoxide, LOOH, while regenerating LO_2_^•^. The chain terminates when two LO_2_^•^ radicals recombine to form diamagnetic products, such as the unstable tetroxide LO_4_L (via the Russell mechanism), which rapidly decomposes into nonradical species.^15,16^ This defines the termination phase (also shown in Figure 1A). The complete lipid peroxidation reaction network is more complex than summarized here, featuring asymmetric recombination pathways and chain-branching reactions that can increase the number of chain carriers from diamagnetic reactants, potentially causing complex kinetics exhibiting pitchfork bifurcation.^11^ These aspects are secondary to the current study, where our focus is on the central symmetric recombination of two LO_2_^•^ radicals. Previous studies have identified this step as a potential site of magnetic sensitivity, due to its compatibility with the RPM.^5,10,17^

**Figure 1:**
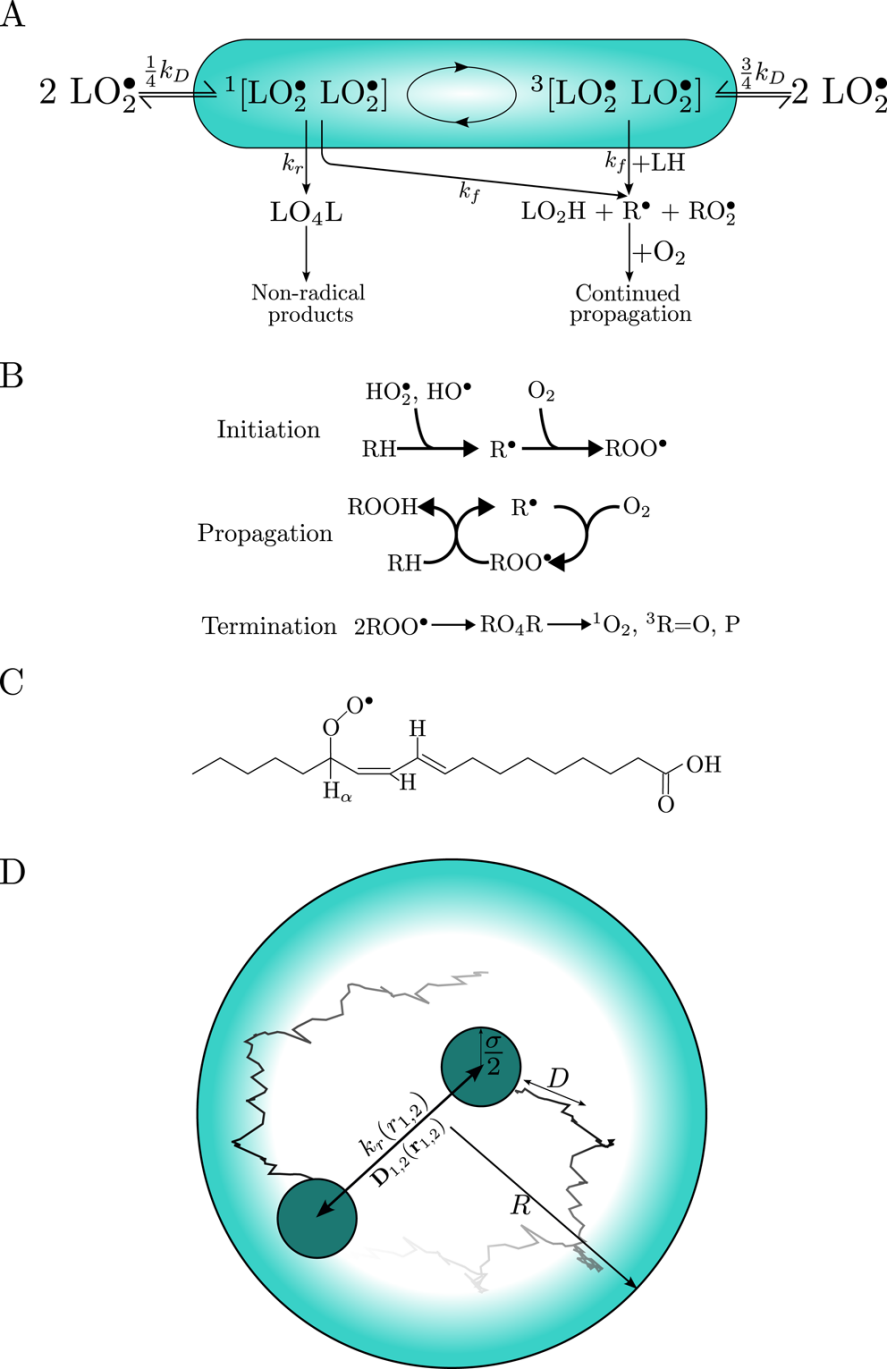
(A) Reaction scheme for lipid F-pairs undergoing singlet–triplet interconversion (represented by the circulating arrows), the key reaction channels, and the associated rates. (B) Steps involved in lipid peroxidation reactions, with each step labeled. (C) Graphical representation of a peroxyl radical. (D) Schematic representation of the system modeled in the Brownian dynamics simulations: A pair of disc-like lipid radicals of radius σ/2 constrained to a 2D circular microdomain of radius *R*, diffusing with diffusion coefficient *D*. Both radicals recombine with distance-dependent rate constant *k*_r_, and are subject to inter-radical couplings such as the EED interaction.

The RPM is thought to facilitate a plethora of magnetosensitive biological phenomena, most notably the avian magnetic compass.^18,19^ At the heart of the RPM is the principle that the spin dynamics of transient radical pairs can be influenced by external magnetic fields, thereby modulating the yield of reaction products derived from singlet (S) and triplet (T) electronic spin states.^20^ Figure 1A illustrates the proposed reaction scheme for a pair of peroxyl lipid peroxyl radicals. Upon diffusive encounter, the two radicals are assumed to have uncorrelated spin states, forming an S state in one-quarter of the encounters and a T state in the remaining three-quarters, i.e., an F-pair. Accordingly, the rates of formation are 1/4 *k*_D_ for singlet encounters and 3/4 *k*_D_ for triplet encounters, where *k*_D_ denotes the diffusion-limited encounter rate. Recombination is spin-permitted from the singlet state, enabling the direct formation of diamagnetic products through bond formation. In contrast, recombination from the triplet state is spin-forbidden and thus requires intersystem crossing to a singlet configuration. If spin relaxation and decoherence are sufficiently slow, then such intersystem crossing can occur through coherent spin evolution driven by internal magnetic interactions, most notably hyperfine couplings with nuclear spins. Under these conditions, the spin dynamics become sensitive to weak external magnetic fields through the Zeeman interaction, which alters the energy levels and modulates the singlet–triplet interconversion. This coherent evolution is schematically represented by circular arrows in Figure 1A. The external magnetic field thereby affects the branching ratio between the S and T populations, ultimately biasing the probability of recombination versus continued radical propagation. Only radical pairs in the singlet state can recombine to terminate the chain reaction. In contrast, both the S and T states can lead to the continuation of the lipid peroxidation chain by generating a new lipid radical (L^•^), which can react again with molecular oxygen to regenerate a lipid peroxyl radical (LO_2_^•^). Eventually, such a radical might encounter another radical, forming a new radical pair and perpetuating the process. In summary, when the spin dynamics of lipid peroxyl radical pairs is magnetosensitive, the overall rate and extent of lipid peroxidation can become responsive to weak magnetic fields.

The hypothesis that radical pairs could underlie magnetic field effects (MFEs) in lipid peroxidation has long been a topic of debate.^21^ Early theoretical analyses argued that the symmetric recombination of two lipid peroxyl radicals (LO_2_^•^) could not exhibit MFEs due to the presumed insignificance of hyperfine couplings and the identical *g*-factors in both radicals, thereby ruling out hyperfine and Δ*g*-driven S/T interconversion.^11,22^ As a result, those studies focused on the much less prevalent asymmetric recombination of LO_2_^•^ with lipid radicals L^•^. This view was later challenged by Sampson et al., who identified a substantial hyperfine coupling involving the α-proton in LO_2_^•^, thereby reviving the plausibility of MFEs on the more common symmetric recombination pathway of lipid peroxyl radicals.^10^ In addition, MFEs on the reaction of molecular oxygen with L^•^ have also been considered in the doublet-triplet pair framework.^23^ While these studies support the theoretical possibility of MFEs arising from the RPM in lipid peroxyl radical recombination, their practical feasibility remained uncertain due to the unknown extent of spin relaxation in LO_2_^•^ radicals within membrane environments. A partial resolution was recently offered by Grüning et al., who demonstrated that *intraradical* spin relaxation is not necessarily detrimental to MFEs in these systems.^14^ In particular, hyperfine-induced and spin-rotational relaxation mechanisms were found to be negligible. The dominant intrapair relaxation pathway was instead attributed to modulation of the anisotropic *g*-matrix caused by molecular tumbling within the membrane, which becomes significant only at high magnetic fields. This leaves open the possibility of MFEs at low fields. However, the study did not account for *inter*-radical interactions, especially the ubiquitous electron–electron dipolar (EED) coupling and its relaxation-inducing modulation via thermal motion. This omission limits the predictive scope of the findings, as inter-radical couplings, such as the EED interaction, are known to suppress MFEs under static conditions in weak fields, and their thermally driven modulation introduces a potentially significant but previously uncharacterized relaxation channel.

The two most common inter-radical interactions, the exchange and EED interactions, generally suppress MFEs in weak fields because, as dominant interactions, they energetically separate S and T states, which are the eigenstates of these interactions, thereby suppressing coherent S/T interconversion via weak hyperfine interactions.^24,25^ This is a general theme, not limited to MFEs in lipid peroxidation, but applies whenever radical pairs are immobilized at close distances, as is often the case for radical pairs in biological systems.^24,26^ To overcome this predicament, we have previously suggested that magnetosensitivity could be recovered if the system were extended to a radical triad, in which case EED interactions with the third radical can drive S/T interconversion in an encounter pair.^25,27^ In fact, MFEs in lipid peroxidation were predicted to be enhanced in the presence of a third radical.^10^ However, if reliant on three-radical encounters, this can only manifest in scenarios of high radical concentration, possibly in events such as ferroptosis, which are associated with spikes in ROS.^10^ Could magnetosensitivity in weak fields also endure in radical pairs? The presumption that this is actually possible is provided by the recent study of radical pair magnetoreception in birds by us and others, which showed that the quantum Zeno effect^28,29^ may be leveraged to ameliorate the deleterious effect of inter-radical interactions in isolated radical *pairs*.^24^ The scenario here is, however, more complex than that used in^24^ as the radical pair is subject to significant diffusive displacement, giving rise to potentially many re-encounters prior to recombination.

Inter-radical interactions are distance-dependent in their strength. While the exchange interaction is steeply peaked at contact, the EED interaction extends considerably further outward due to its comparably weak *r* ^–3^ decay with the inter-radical distance *r*. Therefore, the diffusive motion of the lipid radicals constantly fluctuates the strength of these interactions.^30^ This is a potential cause of quantum decoherence, which threatens to curtail these magnetosensitive dynamics, leading to the loss of MFEs.^25^ Therefore, including the static and dynamic effects of the EED interaction on the reaction outcomes is imperative if we are to realistically assess MFEs in lipid peroxidation. A recent treatment of Lukzen et al. in fact closely approaches this ideal by realizing a simulation in the framework of the integral encounter theory applied to radical particles diffusing in two dimensions.^31^ Although their results demonstrate marked magnetosensitivity, they employed a model truncating the EED coupling under the secular approximation and applying the magnetic field perpendicular to the membrane plane (such that the dipolar coupling interaction depends in the laboratory frame on distance alone). As this secular approximation strictly applies to high fields only, the question of RPM-based magnetosensitivity in lipid peroxidation remains. Here, we attempt to provide a more conclusive evaluation for confined lipid peroxyl radicals subject to static and fluctuating EED at any magnetic field. We achieve this by simulating the translational diffusion of a model lipid radical pair through Brownian dynamics, which then informs all-encompassing spin dynamics calculations. Thus, this contribution aims to address the important open question: can the radical pairs implicated in lipid peroxidation be magnetosensitive despite the dynamic, strong inter-radical coupling from the Brownian diffusive motion of the radical partners in the lipid membrane? And, if so, is the quantum Zeno effect central to this?

## Theory

We model the evolution of the spin density operator, ρ̂(*t*), accounting for electronic and nuclear spin degrees of freedom of the radical pair, using a master equation framework as proposed in^32^. In this formalism, the spin dynamics arise from the interplay of three key processes: coherent spin evolution, spin-selective radical pair recombination, and spin relaxation induced by the thermal motion of the radical pair. The equation of motion can be represented as (ℏ = 1)

ddtρ̂(t)=L^^ρ̂(t)+R^^ρ̂(t)=−i[Ĥ,ρ̂(t)]−{K̂,ρ̂(t)}+R^^ρ̂(t) 1

where [·, ·] and {·, ·} denote the commutator and the anticommutator, respectively, *Ĥ* is the time-averaged Hamiltonian, and *K̂* the time-averaged recombination operator. These quantities are averaged over the Brownian dynamics trajectory of the translational diffusion of the radical pair, which modulates the inter-radical displacement and is described in detail at the end of this section. R^^ is the relaxation superoperator that models spin relaxation induced by modulation of Hamiltonian parameters by thermal motion, which is discussed in further detail below. The effects of coherent evolution and recombination are subsumed in the Liouvillian L^^ for later reference.

The time-averaged spin Hamiltonian *Ĥ* induces the coherent motion and comprises the hyperfine interaction of the electron spin with the α-proton in LOO^•^, the electron–electron dipolar interaction, and the Zeeman interaction, as given by

2a $${Ĥ}_{\mathrm{HFC}}=\sum_{i=1,2}a_{\mathrm{iso}}{Î}_{i}\cdot {Ŝ}_{i}$$

2b $${Ĥ}_{\mathrm{dipolar}}={Ŝ}_{1}\cdot D_{1,2}\cdot {Ŝ}_{2}$$

and

2c $${Ĥ}_{\mathrm{Zeeman}}=\mu_{B}\sum_{i=1,2}{Ŝ}_{i}\cdot g\cdot B$$

respectively. Here, **Ŝ**_*i*_ and **Î**_*i*_ denote the spin operators for the electron and the only significant nuclear spin in radical *i* ∈ {1, 2}, respectively. *a*_iso_ is the isotropic hyperfine coupling constant, **g** is the electron *g*-tensor, and **D**_1,2_ denotes the electron–electron dipolar coupling tensor, averaged over the radical pair trajectory, that is, the average of **D**_1,2_(**r**_1,2_(*t*)) ≡ −μ_0_ *g*_*e*_^2^μ_*B*_^2^/(4π*r*_1,2_^3^) (3**e**_1,2_ **e**_1,2_^*T*^–**1**) with **r**_1,2_ denoting the displacement vector of radicals 1 and 2, *r*_1,2_ ≡ |**r**_1,2_|, **e**_1,2_ = **r**_1,2_/*r*_1,2_, and **1** representing the identity matrix of dimension 3 × 3. Other variables have their canonical meaning. All Hamiltonian terms are assumed in angular frequency units; we explicitly divide by (2π) when referring to linear frequencies.

The chemical reactions of the radical pair are accounted for by the reaction operator

3 $$\mathrm{K̂}=\frac{k_{r}}{2}{\mathrm{P̂}}_{S}+\frac{k_{f}}{2}\mathrm{1̂}$$

where *k*_r_ is the time-averaged singlet recombination rate constant, and *k*_f_ describes spin-independent reactions of the radical pair that lead to chain propagation, such as hydrogen-abstraction from LH. *P̂*_S_ is the singlet projection operator, and 1̂ stands for the identity operator. The recombination rate constant is obtained from the Brownian dynamics simulations by averaging an exponential model of the intrinsic, distance-dependent rate constant, *k*_r_(*r*_1,2_) = *k*_r,0_ exp­(−β­(*r*_1,2_–σ)), over long-time trajectories, where β is a characteristic decay parameter and σ is the contact distance of the radicals.

In order to incorporate relaxation processes into simulations involving rapid recombination (the quantum Zeno regime^24,33,34^ ), we follow the suggestion of Fay et al.,^32^ using an approach derived from the Nakajima–Zwanzig master equation in the Schrödinger picture (unlike the Redfield relaxation tensor, which results from an analogue approach in the interaction representation), contained within the relaxation superoperator R^^ .^35,36^ Despite also being of second order in the system-bath interaction, the Nakajima–Zwanzig approach has proven considerably more accurate than Redfield theory when applied to reaction yields, as it accommodates non-Markovian effects to second order and appears to generally cope better with stronger system-bath coupling.^32^ For Hamiltonian fluctuations described by *H*_1_ = ∑_*i*_ *X*_*i*_(*t*)*Â*_*i*_, where *X*_*i*_ is a random variable reflecting the lipid radicals’ motion and coupling to the spins (⟨*X*_*i*_⟩ = 0), and *Â*_*i*_ is an operator in the system Hilbert space, R^^ can be written as

4 R^^=−∑j,k∫0∞dτgj,k(τ)A^^j†eL^^τA^^k

where *g*_*j,k*_(*t*) is the time correlation function of *X*_*j*_ and *X*_*k*_ such that

5 $$g_{j,k}\left(t\right)=\left\langle X_{j}^{*}\left(0\right)X_{k}\left(t\right)\right\rangle$$

and A^^i represents the commutation superoperator associated with *Â*_*i*_, i.e., A^^i·=[Âi,·] . Given that L^^ can be expressed as L^^·=−i(Ĥeff·−·Ĥeff†) with *Ĥ*_eff_ = *Ĥ*–*iK̂*, the equation of motion (eq 1) can be written in the nonunitary eigenbasis of *Ĥ*_eff_, for which eq 6 takes the form of a sum over products of matrix elements of *Â*_*i*_ and *Â*_*j*_ multiplied by spectral densities

6 $$j_{j,k}\left(\omega \right)=\int_{0}^{\infty }\mathrm{dt}g_{j,k}\left(t\right)e^{i\omega t}$$

evaluated at the eigenvalue differences of *Ĥ*_eff_.^37^ Here, the *X*_*i*_(*t*) values are chosen to describe the fluctuations of the elements of the EED interaction tensor and the *g*-matrix (times the magnetic field). We represent the corresponding correlation functions as multiexponential decays, as appropriate for the studied scenario:

7 $$g_{j,k}\left(t\right)=\sum_{n}c_{j,k,n}\;\exp\left(-t/\tau_{n}\right)$$

Here, the sum runs over a set of characteristic decay times, τ_*n*_; *c*_*j*,*k*,*n*_ are expansion coefficients, obtained by fitting the corresponding covariance functions; and ∑_*n*_ *c*_*j,k,n*_ = ⟨*X*_j_^*^ *X*_*k*_⟩ is the covariance (mean squared fluctuation for *j* = *k*) of the system-environment couplings labeled *j* and *k*.

To assess the magnetosensitivity of the lipid peroxide radical pair systems, we evaluate the singlet recombination yield

8 $$\Phi_{S}\left(B\right)=k_{r}\int_{0}^{\infty }\mathrm{dt}\;\mathrm{Tr}\left\{{\mathrm{P̂}}_{S}\mathrm{\rho ̂}\left(t,B\right)\right\}$$

as a function of the applied magnetic field *B* ≡ |**B**|. In this system, a larger Φ_S_ represents a greater degree of lipid chain termination and, by extension, reduced lipid peroxidation. As the recombination yield depends on the field orientation as a result of the directionality of the Zeeman and EED interactions, we average Φ_S_ over 361 magnetic field orientations, produced by uniform sampling of polar and azimuthal angles θ and ϕ. The MFE is then calculated as

9 $$\Delta \Phi_{S}\left(B\right)=\Phi_{S}\left(B\right)-\Phi_{S}\left(0\right)$$

representing the field-induced difference in singlet recombination probability relative to the zero-field case.

## Model

We model the radical pair system as a pair of soft discs (of radius σ/2, where σ denotes the contact distance), undergoing random walks within a circular planar microdomain of radius *R*. In the overdamped limit, the Brownian dynamics of the discs can be described by the stochastic differential equation

10 $$\mathrm{dr}=\frac{F}{\gamma }\mathrm{dt}+\sqrt{2D}\mathrm{dW}$$

where **r** is a supervector of the positions of the two radicals (**r**_1_ and **r**_2_; dimension 4), **F** is the corresponding supervector of forces acting on the discs, and **W** denotes the Wiener process that encapsulates the stochastic motion inherent in these systems. *D* is the diffusion coefficient of the radicals, and the damping constant γ is given by $\gamma =\frac{k_{B}T}{D}$ . **F** may be broken down into individual contributions that describe the forces acting between the radicals, **F**_1,2_ = −**F**_2,1_, and the repulsive forces of the microdomain boundary on the lipid radicals **F**_*i,B*_, *i* ∈ {1,2}. We assume that particles and boundaries interact via a ‘force-shifted’ Lennard-Jones potential,^38^ for which attractive interactions are removed and the repulsive interaction force goes to zero at a critical distance $r_{C}=\sigma {\left(\frac{26}{7}\right)}^{1/6}$ while retaining continuity of the force derivative. Specifically, with the standard Lennard-Jones potential for the two radicals given by

11 $$u_{\mathrm{LJ}}\left(r\right)=4\epsilon \left[{\left(\frac{\sigma }{r}\right)}^{12}-{\left(\frac{\sigma }{r}\right)}^{6}\right]$$

The shifted-forces variation is written:

12 $$u_{\mathrm{SF}}\left(r\right)=\{\begin{array}{ll} u_{\mathrm{LJ}}\left(r\right)-\left(r-r_{C}\right)u_{\mathrm{LJ}}^{\prime }\left(r_{C}\right)-u_{\mathrm{LJ}}\left(r_{C}\right) & \mathrm{if}\;r<r_{C}\\ 0 & \mathrm{if}\;r\geq r_{C} \end{array}$$

as in^38^.

Thus, for the inter-radical force, we obtain **F**_1,2_ = −*u*_SF_^′^(*r*_1,2_)**r**_1,2_/*r*_1,2_ with **r**_1,2_ = **r**_1_–**r**_2_. The forces between the radicals and the edge of the microdomain take the form **F**_*i*,B_ = *u*_SF_^′^(*R*–*r*_*i*_)**r**_*i*_/*r*_*i*_. To evaluate the correlation functions for the spin dynamics simulations, for every set of parameters, we analyzed 4096 individual Brownian dynamics trajectories with a duration of 50 μs each, sampled at a resolution of 0.01 ns. The initial placement of the radicals was random, subject to the constraint that the disc–disc and disc-boundary distances exceeded σ. The stochastic differential equation was propagated using a modified Euler-Heun method with adaptive time stepping based on an error estimator due to Lamba and Rackauckas,^39^ as implemented by the DifferentialEquations.jl package in Julia.^40^ This adaptive time step was capped such that the expected diffusive displacement was not greater than 0.5 Å per time step (*dt*_max_ = *dr*_max_^2^/4*D*). To calculate correlation functions for the dipolar coupling tensor and *k*_r_, the trajectory mean of each observable was subtracted and time-lagged products *X*(*t*)*X*(*t* + τ) were evaluated for all lags τ­(up to 10 μs) and averaged over all 4096 trajectories. Autocorrelation and cross-correlation functions were fitted using a least-squares method to multiexponential decays employing 12 characteristic time constants (logarithmically spaced from 0.1 ns to 2 μs). We set ϵ = 1 *k*_B_ *T* and σ = 9 Å, and explored the dynamics for the typical mutual diffusion coefficients *D* = 0.14 and 0.5 Å^2^ ns^–1^, microdomain radii of *R* = 20, 25, 30, and 35 Å, and isotropic hyperfine coupling constants of *a*_iso_/(2π) = 10.3 and 13.5 MHz. Correlation functions were calculated for the components of the dipolar coupling tensor and the recombination rate constant. Other parameters were taken from various experimental and computational investigations: isotropic hyperfine coupling constants *a*_iso_ were taken from Sampson et al.;^10^ the contact distance σ was calculated assuming a hexagonal packing of cylindrical lipids from the known area per lipid;^10^ diffusion coefficients *D* are in agreement with experimental^41^ and computational^42^ investigations; microdomain radii were chosen in agreement with the definition of the lipid raft domain by Kusumi et al. in^43^. The *g*-matrix and parameters that define Δ*g*-relaxation were taken from molecular dynamics and DFT analysis of this system by Grüning et al.^14^ By varying *D*, *R*, and *a*_iso_, a wide range of realistic scenarios is covered.

Figure 2 shows a flowchart schematic of the end-to-end simulation workflow: from the simulations of all 4096 Brownian trajectories; evaluation of time-dependent parameters; evaluation of these parameters’ covariance functions and their multiexponential fits; evaluation of spectral densities in order to form the relaxation superoperator R^^ ; solution of the master equation to find ρ­(*t, B*, θ, ϕ) for every magnetic field orientation defined by angles θ and ϕ; evaluating yield variability and averages across the range of possible system parameters investigated here.

**Figure 2:**
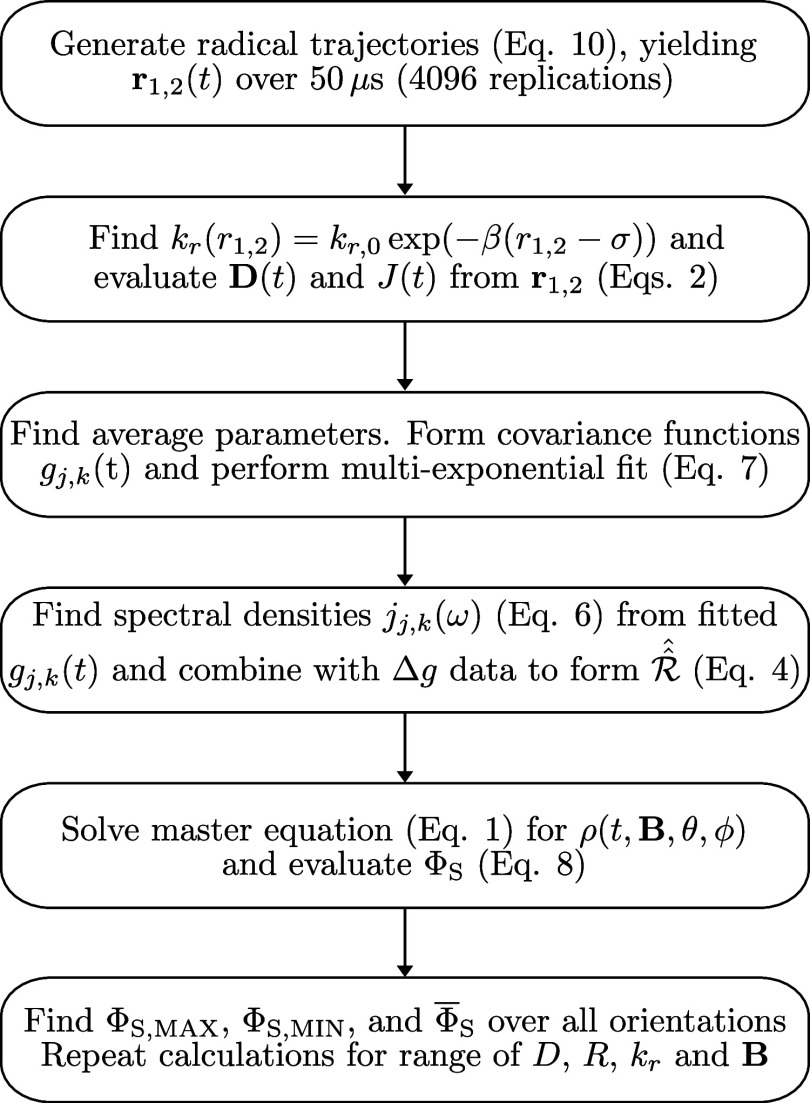
Schematic showing the key steps of our approach. We use a system of stochastic differential equations to model the motion of the radical pair in a microreactor, as depicted in Figure 1D, from which is obtained the time-dependent inter-radical vector **r**_1,2_(*t*). The momentary dipolar coupling tensor **D**_1,2_(*t*) is evaluated using eq 3, and the instantaneous recombination rate constant obtained from *k*_r_(*t*) = *k*_r,*0*_ exp­(−β­(*r*_1,2_(*t*)−σ)); the momentary exchange coupling *J*_Ex_(*t*) = *J*_Ex,0_ exp­(−β­(*r*_1,2_–σ)) is evaluated in an analogous fashion. The covariance functions of these quantities determine the spin relaxation superoperator R^^ via their spectral densities, which here are derived from multiexponential fits of the various covariance functions (eqs 8 and 9). Δ*g*-relaxation is incorporated using parameters from.^14^ This, along with the system Liouvillian L^^ , forms the equation of motion (eq 1) evolving the density matrix ρ̂(*t*). The singlet yield for each orientation on a grid of orientations is calculated using eq 10, from which the maximum, minimum, and average values are calculated. This process is repeated for a range of recombination rate constants *k*_r_, magnetic field intensities *B*, diffusion coefficients *D*, microdomain radii *R*, hyperfine couplings *a*_iso_, forward reaction rate constants *k*_f_, and exchange coupling constants *J*_Ex_ to form a broad assessment of variable system properties.

## Results

We performed Brownian dynamics simulations of the translational diffusion of lipid peroxyl radical pairs constrained to circular, planar regions, referred to here as microdomains, as schematically illustrated in Figure 1D. Simulations were conducted using typical lipid peroxyl diffusion coefficients (*D* = 0.14 and 0.5 Å^2^ ns^–1^) across a range of microdomain radii (*R* = 20, 25, 30, and 35 Å). We refer the reader to the Discussion section for a detailed discussion of the rationale and biological implications of this spatial confinement. For present purposes, it suffices to note that confinement promotes radical re-encounters, which are essential for generating sizable MFEs.

The translational Brownian motion of the radical pair dynamically modulates both the inter-radical distance and the relative orientation. These variations in turn affect the spin dynamics by altering the EED coupling as well as the spin-selective recombination rate constant. We incorporate these effects within a master equation framework, where their influence manifests through averaged quantities: the mean spin Hamiltonian governing coherent evolution, averaged recombination rate constants, and a relaxation superoperator capturing decoherence induced by stochastic fluctuations. The latter is introduced in eq 6 and depends on the covariance functions of the modulated spin-interaction parameters. These covariance functions were extracted from long-time scale Brownian dynamics simulations. Due to the rotational averaging in the plane, assumed to be the *x*–*y* plane, the mean EED coupling tensor is diagonal, with *D*_*XX*_ = *D*_*YY*_ (with the spin indices suppressed in the EED coupling parameter for brevity). Furthermore, the fluctuating components of the EED tensor are characterized by five independent covariance functions, including the autocovariances of *D*_*XX*_, *D*_*XY*_, and *D*_*ZZ*_, as well as the cross-covariances between *D*_*XX*_ and *D*_*YY*_, and between *D*_*XX*_ and *D*_*ZZ*_. The remaining components follow from symmetry.

The spin dynamics were simulated using these Brownian-dynamics-derived covariance functions. The initial spin state of the radical pair was chosen as a pure triplet, ρ(0) ∝ *P̂*_*T*_ = 1̂–*P̂*_*S*_, consistent with prior studies.^10,14^ While radical pairs typically form as uncorrelated F-pairs, this approximation effectively captures the relevant spin dynamics. In initial encounters, approximately one-fourth of the pairs form singlet states and recombine immediately, leaving the triplet subpopulation to drive the subsequent magnetosensitive dynamics. Although this initial singlet recombination adds a field-independent background to the recombination yield, thereby reducing the relative magnitude of the MFE, the field-dependent trends remain similar for both the F-pair and triplet initial conditions, provided that the singlet recombination is rapid, as assumed here. This point is explicitly demonstrated in Figure S7 of the Supporting Information (SI).

To ensure the generality of our conclusions, we explored a broad parameter space. In addition to varying the microdomain radius (*R*) and diffusion coefficient (*D*), we also investigated two values of the isotropic hyperfine coupling constant (*a*_iso_), representing two isomers of the linoleic acid peroxyl radical. The full results of this parameter survey are presented in the SI (see Figure S1) and reveal qualitatively consistent behavior across all systems studied. For the sake of clarity and conciseness, we focus hereafter on a representative system, termed the baseline system, defined by *R* = 25 Å, *D* = 0.14 Å^2^ ns^–1^, and *a*_iso_/(2π) = 10.3 MHz, corresponding to the 13ze isomer. Typical effective correlation times for this system are 0.13 μs for the *D*_*XX*_ covariance and 0.16 μs for the *D*_*ZZ*_ covariance.

We simulated the magnetic field dependence of the singlet recombination yield, Φ_S_, as a function of the effective recombination rate constant, *k*_r_. This effective rate reflects the average over all sampled radical pair configurations and is, due to diffusive paths, reduced relative to the intrinsic contact recombination rate *k*_r,0_. The extent of this reduction increases with both the diffusion coefficient (*D*) and the domain radius (*R*) as a greater mobility and spatial freedom lower the probability of close encounters. For the baseline system, we find *k*_r_ ≈ 0.02 *k*_r,0_.

Figure 3 presents line plots of Φ_S_ as a function of magnetic field strength, *B*, for three representative recombination regimes: (i) slow recombination (*k*_r,0_ = 10 μs^–1^; yellow), (ii) symmetric recombination (*k*_r,0_ = 49 μs^–1^; pink), and (iii) fast recombination (*k*_r,0_ ∼ 1000 μs^–1^; purple), where the exact value was chosen to maximize the high-field MFE. The “symmetric” case refers to the condition where the recombination rate constant is comparable to the rate constant of the competing forward reaction (*k*_r_ ≈ *k*_f_), a scenario generally viewed as auspicious for large MFEs due to balanced kinetics. Each regime is shown in both the absence (panel A) and the presence (panel B) of spin relaxation arising from fluctuations in the EED interaction. Across all cases, we observe that increasing the recombination rate enhances the magnetic field sensitivity of the recombination yield even in the absence of relaxation. This behavior can be attributed to the quantum Zeno effect and the associated alteration of the spectrum of the effective spin Hamiltonian ( H^eff *H*_eff_). More specifically, fast recombination leads to quasi-degeneracy between triplet sublevels and the recombining manifold, making them more susceptible to magnetic perturbation, even by weak fields, when a suitably orientated applied magnetic field facilitates coupling between these nearly degenerate states. This mechanism, previously discussed in the context of triplet-born radical pairs in cryptochrome, is also applicable here (see^24^ for details).

**Figure 3:**
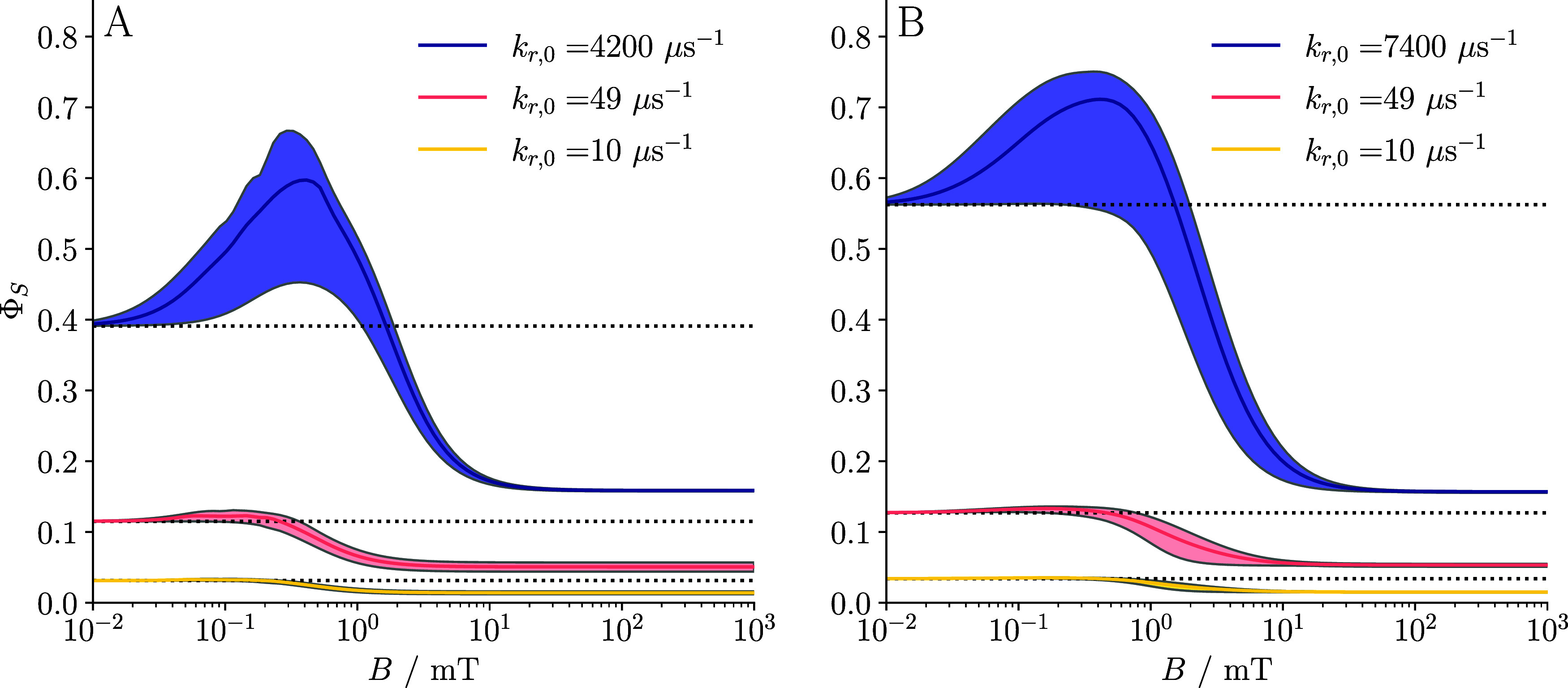
Plots of Φ_S_ against applied magnetic field *B* for three different singlet recombination rate constants corresponding to “slow” (*k*_r,0_ = 10 μs^–1^, yellow lines) and “symmetric” radical recombination (*k*_r,0_ = 49 μs^–1^, corresponding to *k*_r_ = 1 μs^–1^, pink lines), and the quantum Zeno regime (purple lines) in (A) the absence and (B) presence of EED-induced spin relaxation. Dotted lines represent zero-field yield Φ_S_(0). For the quantum Zeno regime, the chosen *k*_r,0_ values indicated in the legend maximize ΔΦ_S_(*B*) (deviation of solid lines from dotted lines) in the high-field limit. Solid lines indicate the mean value of Φ_S_, and the plot is shaded between lines that show the minimum and maximum values of Φ_S_ over a directional sweep of the magnetic field at each value of *B*. The system modeled here is the baseline system with *R* = 25 Å, *D* = 0.14 Å^2^ ns^–1^, *a*_iso_/(2π) = 10.3 MHz, and *k*_f_ = 1 μs^–1^. Note that combining EED interactions with fast spin-selective recombination boosts the magnetic field sensitivity via the quantum Zeno effect. In addition, dipolar relaxation enhances the singlet recombination yield in the low-field region, thereby boosting the MFE in high fields. Overall, this indicates that EED interaction can actually be an asset to the magnetosensitivity.

The shaded regions in Figure 3 capture the directional dependence of the recombination yield, showing the range of Φ_S_ values that result from variation of the orientation of the magnetic field relative to the membrane. The solid curves represent the mean values over all orientations. Directional sensitivity is particularly pronounced in the low-field regime for fast recombination, consistent with the Zeno mechanism: at low fields, subtle changes in magnetic orientation can strongly influence the dynamics, in line with the outlined mechanism. In contrast, at high fields, the Zeeman interaction dominates over EED, energetically separating the |*T*_+_⟩ and |*T*_–_⟩ states, thereby restricting spin evolution to the *S*/*T*_0_ manifold and reducing the anisotropy.

A comparison between panels A and B reveals that EED-induced relaxation attenuates low-field MFEs in the slow and symmetric recombination regimes and shifts the characteristic half-saturation field toward higher values. However, in the fast recombination regime, relaxation enhances the singlet–triplet interconversion in low fields, thereby increasing the singlet yield in low fields and amplifying ΔΦ_S_(*B*) in high fields. Regarding the low-field response, although EED-mediated relaxation alters the shape of the MFE response curve, the overall magnetic sensitivity remains relatively robust for a large *k*_r_. It may even be slightly enhanced in the very-low-field region (although clearly the MFEs are generally small at such low fields). However, it should be noted that optimal sensitivity under these conditions requires faster recombination kinetics to offset the decoherence-inducing spin relaxation.

For the baseline system, Figure 4 shows heatmaps of ΔΦ_S_ as a function of the applied magnetic field (*B*) and the recombination rate constant, expressed in terms of *k*_r_ (left axis) and *k*_r,0_ (right axis). While providing a systematic assessment and quantification of MFEs, the qualitative characteristics are fully in line with the behaviors discussed above for the recombination yield Φ_S_. Specifically, we notice that the magnetosensitivity is weak for the “symmetric” recombination regime, realized for *k*_r,0_ = 49 μs^–1^, and even more so for slower recombination. Despite some motional averaging of the EED interaction, the EED interaction is significant in suppressing the magnetosensitivity in this regime. However, increasing the recombination rate constant beyond the “symmetric” regime induces the quantum Zeno effect in triplet-born radical pairs, which significantly increases the MFEs. Including again the relaxation induced by modulation of the EED interaction by the diffusive motion of the radical pairs in Figure 4, panel B, shows that the MFEs are robust to its inclusion in weak fields and can profit from it in the high field. This is a consequence of it inducing S–T transitions in the low field while not strongly affecting the high-field yields, thereby enhancing the overall yield contrast, as discussed above. The overall largest magnetosensitivity is achieved for fast recombination rate constants, on the order of *k*_r,0_ ≃ 10^4^ μs^–1^, suggesting that the quantum Zeno effect, activated by these faster rates, is required to extract the greatest magnetosensitivity available, and this holds true both in lower and higher ranges of magnetic field intensity. The fact that even faster recombination rates than these are not increasingly beneficial to magnetosensitivity results from the eigenvalue spectrum of the effective Hamiltonian,^24^ in particular the quantum Zeno effect rendering the effective spin-selective recombination of the relevant states too slow to impact on the spin dynamics during the radical pair lifetime.

**Figure 4:**
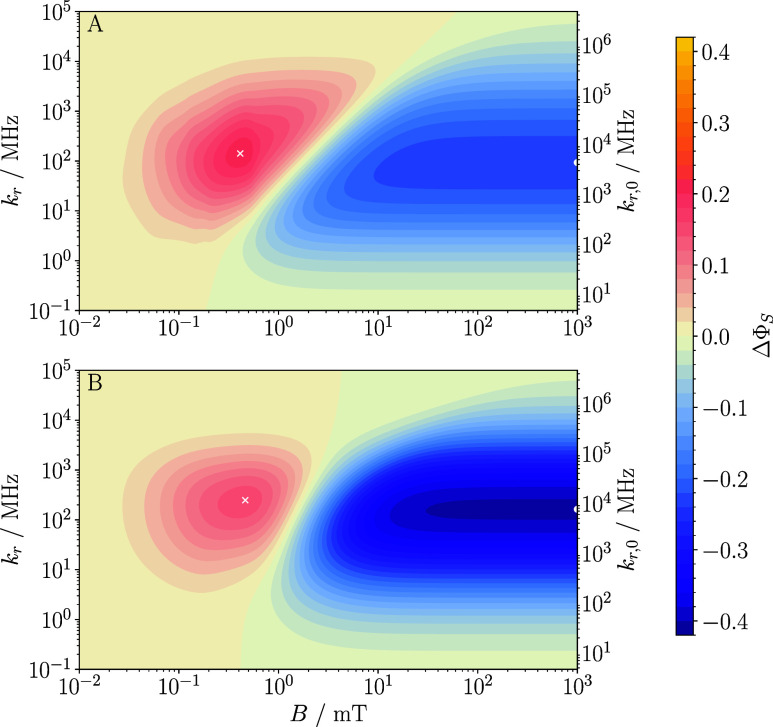
Heatmaps of the absolute difference in singlet yield ΔΦ_S_ as a function of *k*_r_ (left axis), *k*_r,0_ (right axis), and *B* (abscissa) for our ‘baseline system’ (triplet initial state, *R* = 25 Å, *D* = 0.14 Å^2^ ns^–1^). Units of *k*_r_ and *k*_r,0_ are MHz, equivalent to μs^–1^. Panel (A) shows the system’s behavior in the absence of spin relaxation, whereas panel (B) includes spin relaxation induced by fluctuations in EED couplings. Evidently, inclusion of the dipolar relaxation in the quantum Zeno regime is not detrimental to the magnetosensitivity and can even facilitate an enhancement of ΔΦ_S_ in the ‘high field’ scenario. For ease of comparison, the color bar is shared between panels. The white cross and white dot mark maximal low-field and high-field MFEs as assessed via ΔΦ_S_. EED-induced spin relaxation shifts these points of maximal effect to larger values of *k*_r_.

Above, we aimed to elucidate clearly the effects of EED-induced spin relaxation on weak-field magnetosensitivity, which had been neglected in previous assessments. However, the intraradical motion and tumbling in the membrane induce relaxation too. Grüning et al. have recently quantified these intraradical relaxation channels using molecular dynamics trajectories and density-functional theory calculations to inform the required covariance functions of the coupling parameters.^14^ Assessing hyperfine-induced, spin-rotational, and Δ*g*-relaxation, the authors concluded that only the latter, scaling quadratically with the magnetic field intensity, contributes significantly in strong magnetic fields; hyperfine and spin-rotational relaxation were found to be insignificant. We therefore continue our exploration by including Δ*g*-relaxation.

Figure 5 shows selected simulations of the recombination quantum yield as a function of the applied magnetic field (*B*) for a model including the motion-averaged *g*-matrix and Δ*g*-relaxation based on the covariances and effective correlation times derived in^14^. Panel A gives the results for a model where all influences including hyperfine and EED interactions in the coherent evolution and EED-induced spin relaxation have been removed to isolate the effects of Δ*g*-relaxation, while in panel B, interactions include those in Figure 3B in addition to *g*-anisotropy and Δ*g*-relaxation. The most notable feature of these results is that Δ*g*-relaxation provides an efficient S/T interconversion pathway, which in combination with efficient singlet recombination increases singlet recombination yield in high fields. This adds an MFE contribution to the picture, conventionally referred to as the ‘relaxation mechanism’, which opposes the hyperfine-induced S/T interconversion seen above, i.e., it increases the recombination yield in high-field rather than attenuating it. The results in panel B are in good agreement with an analytical result for a limiting case presented in the SI.

**Figure 5:**
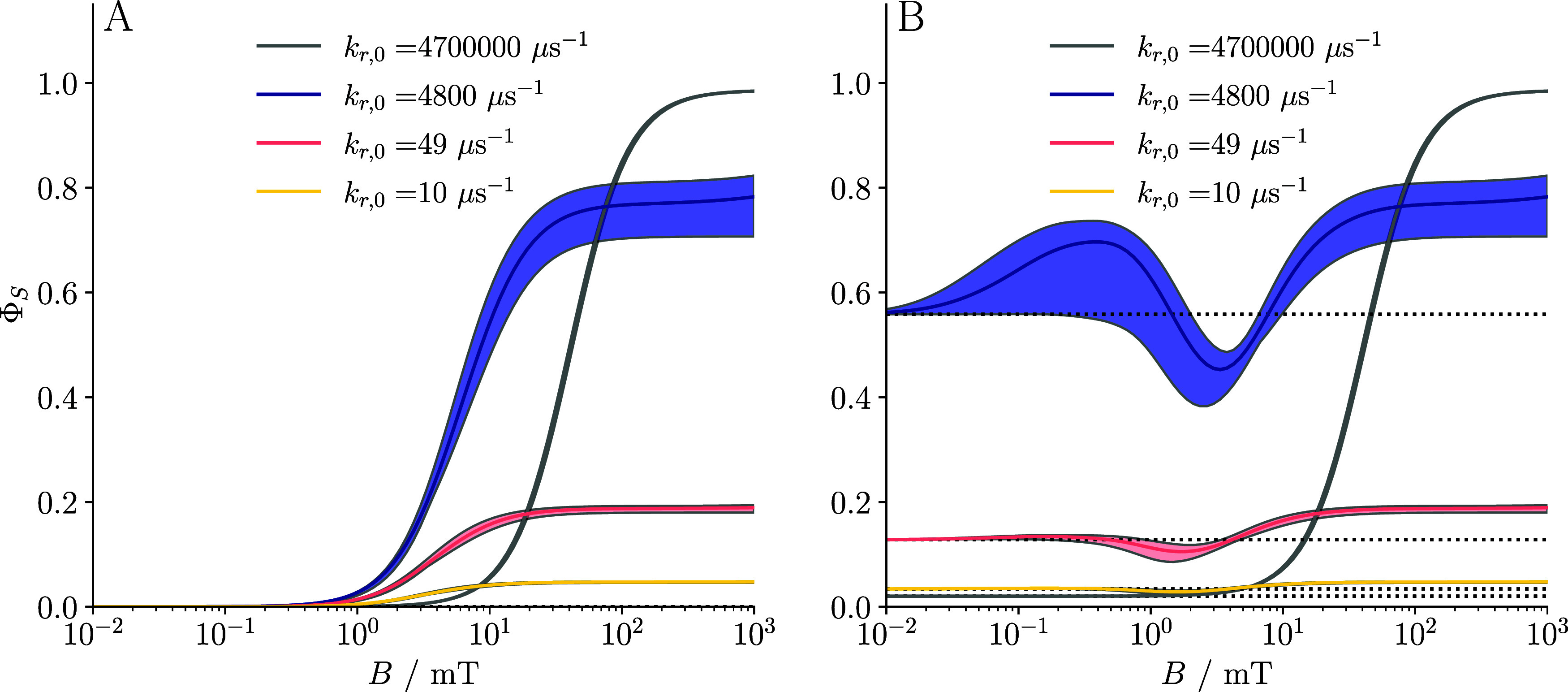
Plots of Φ_S_ against applied magnetic field intensity (*B*) for the “baseline system” for selected singlet recombination rate constants *k*_r,0_, now including Δ*g*-relaxation. Panel (A) shows the result of removing all interactions except for the static Zeeman interaction and Δ*g*-relaxation. Panel (B) includes all effects from Figure 3B in addition to *g*-anisotropy and Δ*g*-relaxation. The following recombination scenarios have been considered: slow recombination (*k*_r,0_ = 10 μs^–1^, yellow lines); symmetric radical recombination (*k*_r,0_ = 49 μs^–1^, pink lines, corresponding to *k*_r_ ≃ 1 μs^–1^); fast Zeno regime recombination (*k*_r,0_ = 4800 μs^–1^; purple lines); and extremely fast recombination (*k*_r,0_ = 47,00,000 μs^–1^, corresponding to *k*_r_ ≃ 1,00,000 μs^–1^, the upper limit of rate constants considered; grayed out). Dotted lines represent the zero-field yield Φ_S_(0). Other details are given in Figure 3. Panel (A) illustrates the predicted large MFEs at high *B* strengths, and negligible low field effects, while Panel (B) shows increased low field effects (particularly for *k*_r,0_ = 4800 μs^–1^, where the quantum Zeno effect is active) and competition between the hyperfine mechanism and Δ*g*-relaxation.

Figure 6 shows heatmaps of the singlet recombination yield, Φ_S_, and the associated MFEs for the “baseline system” including both EED and Δ*g*-relaxation and all pertinent interactions (i.e., the same system as in Figure 5B), representing a full survey over *k*_r_ and *B* for the most comprehensive model investigated here. The MFE in panel B is clearly recognized to arise from the competition of the hyperfine mechanism, sustained in the presence of unavoidable EED interactions and their fluctuations by the quantum Zeno effect, and the relaxation mechanism. This dichotomy of mechanisms is even more evident in the actual recombination yield, presented in panel A. Although huge MFEs are predicted in the high-field/fast recombination limit, these might not be practically accessible as a consequence of limited intrinsic reactivity, i.e., *k*_r,0_. The most striking feature, instead, is the persistence of relatively high magnetosensitivity for moderate to high *B* for *k*_r_ values that despite exceeding the “symmetric” recombination situation are not excessive. Although there is a smaller absolute effect in terms of ΔΦ_S_ than seen in Figure 4B, the effect in this region is still encouragingly strong and is indicative of the possibility of magnetosensitive radical pair dynamics under conditions that are relevant for biological lipid bilayer systems. What is remarkable is that, controlled by *k*_r_, varied magnetic-field-sensitive behavior can ensue, including scenarios featuring low-field effects or abolition of magnetosensitivity. Specifically, it appears feasible to both accelerate and decelerate lipid peroxidation by the judicious application of magnetic fields.

**Figure 6:**
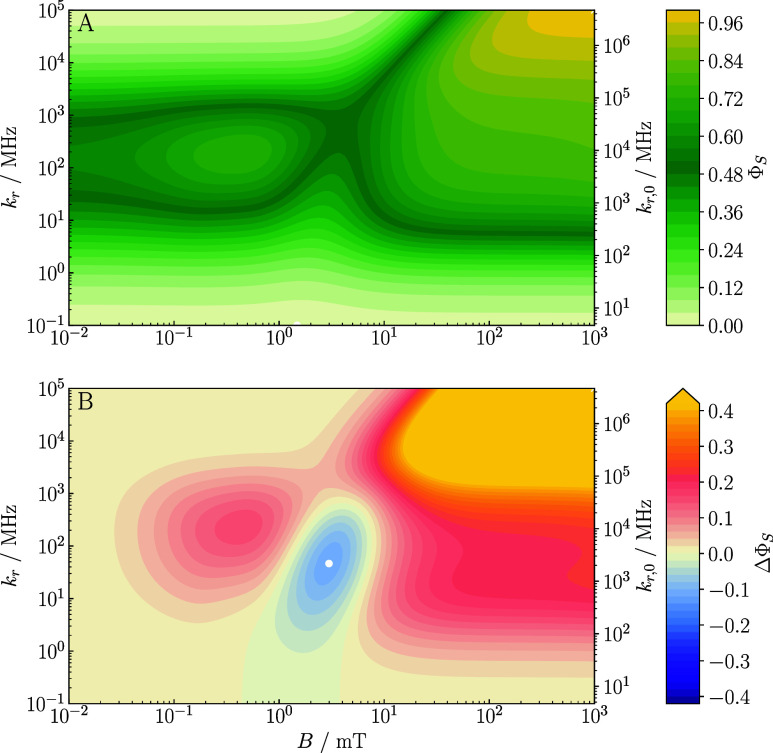
Heatmaps of (A) the singlet recombination yield, Φ_S_(*B*), and (B) the associated MFE, ΔΦ_S_(*B*), as a function of the singlet recombination rate constant, expressed as *k*_r_ (left axis) and *k*_r,_ *_0_* (right axis), and the magnetic field *B* for the “baseline system” with *g*-anisotropy and Δ*g*-relaxation included on top of EED-induced spin relaxation. Units of *k*_r_ and *k*_r,0_ are MHz, equivalent to μs^–1^. Both panels show that resilient MFEs are accessible at moderate field intensities and fast, but reasonable, recombination, and that high-field regions are dominated by Δ*g* effects. See Figure 4 for further details. Corresponding plots for the system with Zeeman interaction and Δ*g*-relaxation only are provided in Figure S8.

## Discussion

The idea that lipid peroxidation might be sensitive to weak magnetic fields has been frequently reported and speculated upon.^5–11^ However, the lack of a robust theoretical foundation, combined with the general difficulty of unambiguously detecting MFEs, particularly within complex biological environments, has hindered progress in this area. The proposition that these effects could arise via the RPM is therefore compelling: it offers a well-defined theoretical framework and situates the phenomenon within the broader context of spin chemistry, where MFEs have been consistently observed in a variety of radical-based chemical reactions.

The question of whether the recombination of the peroxyl radical in lipid membranes could be magnetosensitive due to the RPM has seen important developments recently: DFT studies by Sampson et al. showing unexpected lipid radical hyperfine couplings reopened the possibility of MFEs arising, even in the dominant symmetric recombination of lipid peroxyl radicals.^10^ Subsequent investigation by Grüning et al. showed that intraradical relaxation mechanisms are unlikely to preclude MFEs, but did not treat inter-radical effects, most notably arising from EED coupling. This has been treated to some extent in earlier investigations.^10,31^ However, these studies relied on static immobilized radicals or made simplifying assumptions strictly applicable only in high magnetic fields. Consequently, the question of whether weak-field MFEs can emerge in mobile lipid peroxyl radical pairs remained unanswered.

In this work, we demonstrate that MFEs remain feasible, even in the presence of static and fluctuating EED interactions. A key requirement for sustaining MFEs is the maintenance of a sufficiently high effective recombination rate constant *k*_r_. This induces the quantum Zeno effect, which immunizes the spin dynamics of triplet-born radical pairs to the EED interaction, as discussed here and in greater detail in refs 24,26 . Notably, this fortuitous trait also extends to F-pairs, provided that the singlet recombination pathway is highly efficient. In such cases, the dynamics of F-pairs resemble those of triplet-born pairs, allowing a similar magnetosensitivity.

Our simulations reveal that, depending on the recombination rate constant *k*_r_ averaged over the motion, varying MFEs can emerge. These include a low-field effect in the (sub)­millitesla range arising from hyperfine-driven singlet–triplet interconversion (which can be sustained by the quantum Zeno effect), and an opposing high-field effect dominated by Δ*g*-relaxation, as is characteristic for ‘free’ radical pairs. However, the underlying mechanism differs from the conventional RPM scenario: here, the EED interaction plays a central, active role rather than being a purely suppressive factor. Eventually, when recombination becomes very efficient and the Zeno effect itself loses its magnetosensitivity (an outcome illustrated, for example, in ref 24, where an inverted V-shaped dependence of MFE magnitude on *k*_f_ and *k*_r_ is shown), spin relaxation due to orientational fluctuations modulating the Zeeman interaction in the presence of significant *g*-anisotropy becomes the dominant pathway for singlet–triplet interconversion in high magnetic fields. This relaxation-driven mechanism gives rise to MFEs that can oppose those driven by the “Zeno-effected” coherent evolution. As a result, both positive and negative MFEs can emerge in high magnetic fields, with the sign and magnitude of the effect critically dependent on the recombination rate constant *k*_r_. At the crossover point between these competing mechanisms, the high-field MFE can be effectively canceled, isolating a residual low-field enhancement. Overall, these findings suggest that variability in the effective recombination rate constant may result in the diverse magnetic field sensitivities reported in the literature on lipid peroxidation.

The preceding analysis pertains to the behavior and predictions of our model system. We now consider how these findings may relate to experimental reports of magnetic field effects on lipid peroxidation. Although magnetosensitivity in lipid peroxidation has been reported in numerous studies, systematic investigations using simple, tractable model systems remain relatively scarce. One recent applied study investigated the use of static magnetic fields to mitigate oxidative stress injury during tissue and organ thawing. In that work, cryopreservation benefits, presumably due to reduced oxidation, were observed for magnetic fields in the 1–5 mT range, with an optimum at 4 mT.^44^ This aligns closely with our predictions for the low-field MFE regime, assuming a recombination rate constant on the order of hundreds of MHz. Second, an investigation by Martynyuk et al.,^9^ applying pulsed magnetic fields of induction 5, 50, and 500 μT to liposomal suspensions, only observed effects at 500 μT or 0.5 mT, in agreement with the rise in low-field magnetosensitivity shown in Figure 6B. Such results are particularly compelling, as they imply that this system indeed leverages the quantum Zeno effect to elicit low-field magnetosensitivity.

Another notable contribution is the work of Kabuto et al.,^45^ who explored the magnetic field sensitivity of iron-induced lipid peroxidation in mouse brain homogenates and phosphatidylcholine vesicles. They observed a striking suppression of lipid peroxidation within a narrow magnetic field window between approximately 2 and 4 mT, and an absence of any measurable effect at higher fields (up to 300 mT). Upon first inspection, this result does not appear to align with the results shown in Figure 6. However, in further simulations employing longer correlation times for the Δ*g*-relaxation, we found that elongation of the correlation time by a factor >4 can indeed produce a region of MFE-absence at higher magnetic fields. Thus, the described magnetosensitivity is in agreement with an intermediate *k*_r,0_, inducing the quantum Zeno effect, and Δ*g*-relaxation with a correlation time increased by a factor >4 over those estimated from MD of a simple model system. Plots of the type in Figure 6B with lengthened correlation times are presented in Figure S9, indicating the emergence of a region of 0 MFE in high field once correlation time is multiplied by a factor >4. Such an increase in correlation time appears due to the differences in conditions and complex composition of brain homogenates. Additionally, the temperature difference between that used in the MD simulations that produced the correlation times used in our simulations (310 K) and the temperature at which the experiments in^45^ were conducted (298 K) also contributes to this difference. In light of our results, this suggests an effective recombination rate constant *k*_r_ of ≈700 μs^–1^, positioning the spin dynamics near the quantum Zeno–relaxation crossover.

Our results also tally with various experimental results investigating magnetic effects on oxidative stress *in vivo* in the high-field region. Various studies report increases and decreases in oxidative stress, which we interpret as indicative of effective *k*_r_ values that fall above or below the critical range (*k*_r_ ≈ 700 μs^–1^), depending on the specific biological system. For instance, Cecerska-Heryć et al.^46^ reported a reduction in oxidative stress in human plasma samples exposed to a ∼40 mT quasi-static magnetic field, suggesting dynamics in the fast *k*_r_ regime where relaxation-enhanced singlet–triplet mixing dominates. In contrast, Kthiri et al.^47^ observed an increase in oxidative stress in *Saccharomyces cerevisiae* when subjected to a field of 250 mT, indicative of the slow-*k*_r_ regime. These are representative examples; see^48^ for a comprehensive summary of such MFEs.

Assuming that the model elaborated here applies qualitatively to biological membranes, which is supported by constants of behavior over the wide range of parameters surveyed, these results, taken together, suggest that biological membrane systems may reside near the critical *k*_r_ regime, where high-field MFEs vanish, yet small deviations of *k*_r_ in either direction could lead to positive or negative MFEs in the high field, explaining the divergent effects reported in the literature. Crucially, this variability does not need to reflect large differences in the intrinsic recombination rate constant *k*(_r_), but rather in the effective recombination rate constant *k*_r_, which governs the spin dynamics. These differences may arise from variations in diffusion coefficients or spatial constraints of the microdomain within which lipid peroxyl radicals encounter each other, rather than intrinsic chemical reactivity alone.

Given the strong dependence of the MFEs on the effective recombination rate constant *k*_r_ and the observed variability in experimental outcomes, ranging from increased to decreased oxidative stress, we recommend that future studies of MFEs systematically include low-field exposures around 1 mT in addition to higher fields. Ideally, experiments should be designed to probe a strategic range of field strengths (e.g., 1, 10, and 100 mT) selected in accordance with the predicted MFE profiles (see Figure 6) to ensure that no MFE is overlooked inadvertently due to unknown system-specific values of *k*_r_. Constructing such a comprehensive MFE response profile, in combination with theoretical modeling, would allow a more precise inference of the effective *k*_r_ and expected MFE profile. This detailed knowledge is especially relevant for the rational development of medical applications, such as magnetic cryoprotection,^44^ or therapeutic strategies that aim to enhance radiotherapy or chemotherapy by field-induced increases in ROS production (see, e.g.,^30^). The efficacy of such strategies depends critically on realizing maximal MFEs of the right sign and thus requires accurate magnetic field dosing, which in turn hinges on the correct characterization of *k*_r_. The marked biphasic behavior of the MFE is here not only a challenge but also an opportunity because it introduces the possibility of tuned lipid peroxyl recombination to maximize the desired outcome. Furthermore, for surface-exposed membranes, changes in temperature could be used to alter rate constants, facilitating the movement of the system into the desired region in the parameter space. As for effect sizes, the maximal percentage changes in recombination yield, calculated for a F-pair encounter under the assumption of immediate recombination of the singlet fraction, i.e., (0.75 · ΔΦ_S_)/(0.25 + 0.75 · Φ_S_(0)), at magnetic field intensities *B* of 0.1 1, 10, and 100 mT are predicted as 10, 12, 47, and 230%, respectively.

Finally, caution is generally warranted when MFEs are interpreted in complex biological systems. In such contexts, other molecular targets and interaction mechanisms may dominate over lipid peroxidation, or the overall outcome may reflect compensatory physiological responses that obscure the underlying molecular effects. For example, although Kabuto et al.^45^ reported reduced oxidative stress in simplified systems that align with theory, subsequent studies in more complex models exposed to weak extremely low-frequency magnetic fields have observed enhanced oxidative stress instead. These outcomes are inconsistent with a direct effect on lipid peroxyl radical recombination through the RPM and suggest alternative explanations such as systemic overcompensation, dominance of different radical pair pathways, or entirely distinct interaction mechanisms relevant to low-frequency field exposures.

We conclude with a few remarks about the assumptions underlying our theoretical model and possible directions for future research. A central assumption in our framework is that lipid radicals diffuse within a confined, circular microdomain. This choice is driven by both conceptual reasoning and practical considerations. By imposing spatial confinement, we exclude radical pair trajectories in which the radicals permanently separate and, therefore, cannot contribute to recombination-based MFEs. Naturally, this also implies that the predicted MFEs are somewhat amplified relative to those expected in fully unconfined, freely diffusing systems. Although this idealization may be less justified for synthetic or homogeneous membranes, it becomes more appropriate, indeed, more realistic, when modeling biological membranes. The ‘pickets and fences’ model introduced by Kusumi and colleagues describes the plasma membrane as a laterally heterogeneous, partitioned structure, shaped by interactions with the cytoskeleton and transmembrane proteins.^49,50^ This model is supported by high-speed single-particle tracking data and is now widely accepted as a key feature of membrane organization. We note furthermore that even in the case of nominally homogeneous membranes, the microdomain assumption remains a reasonable approximation. The relatively slow diffusivity of lipid radicals in membranes limits their spatial excursions during the relevant time scales. This supports the notion that radical pairs that have met remain in relatively close proximity during the time that MFEs can develop, thereby justifying the use of spatially confined domains in our simulations. In fact, as shown in Figures S1 and S2, varying the domain size does not qualitatively change the predicted MFE trends, further validating this modeling choice. On a related point, the choice to model the lipid radicals as discs within the microdomain was motivated by the fact that structural fluctuations occur on a much faster time scale than the RP spin dynamics: this is a choice that is used routinely in the field of spin chemistry.^14^

In the present work, our aim is to establish how substantial MFEs can arise at the level of an isolated radical pair. However, ongoing lipid peroxidation chain reactions could cause the accumulation of radicals and interactions beyond the primary reaction pair. While it might be expected that further inter-radical dipolar interactions would disrupt coherent spin evolution and suppress MFEs, theoretical studies of dipolarly coupled multiradical systems indicate otherwise. In the static limit, additional radicals can mediate singlet–triplet interconversion via electron–electron dipolar interactions (the D_n_M mechanism), and broken geometric symmetry can enhance these MFEs.^27,51^ However, such effects require relatively high local radical concentrations,^10^ which are unlikely under typical physiological conditions but could occur in extreme (pathological) scenarios such as ferroptosis. As it is our aim to demonstrate that substantial MFEs already arise for an isolated radical pair via the quantum Zeno effect, we restricted our treatment to this minimal model.

We have also omitted other reaction pathways, such as those involving carbon-centered radicals,^11^ for two reasons: the dynamics of lipid peroxyl radicals are expected to dominate the overall dynamics due to their comparatively higher concentration and because different radical pairs are expected to exhibit similar dynamics, therefore not affecting the overall qualitative picture.

We have excluded effects arising from the exchange interaction from the discussion here due to its rapid decay with increasing *r*_1,2_ as the radicals diffuse apart from contact. Results of the inclusion of both static nonzero exchange coupling and fluctuation-induced exchange relaxation are shown in Figure S6, which confirm that exchange coupling does not suppress MFEs for moderate values of *J*_Ex, 0_ in the quantum Zeno regime. An explicit treatment of diffusion-modulated exchange coupling is expected to predominantly induce singlet–triplet dephasing effects, which have previously been shown not to be detrimental to MFEs in the quantum Zeno regime.^24^

We have furthermore only included relaxation via fluctuating EED coupling and Δ*g*-relaxation, omitting other pathways such as hyperfine modulation and spin-rotational relaxation, as these have been shown to cause negligible effects for this system.^14^ Finally, we include a single hyperfine coupling per radical, as it has been shown by Sampson et al. that additional hyperfine couplings are substantially weaker and, therefore, not expected to substantially alter spin dynamics.

In evaluating the effect of the quantum Zeno effect on lipid peroxidation, we found a strong enhancement of the MFE caused by high recombination rate constants (*k*_r_). While the intrinsic reaction rate constants of tetroxide formation from peroxyl radicals in lipids have not yet been elucidated, *ab initio* calculations on alkylperoxyl radicals suggest that tetroxide formation proceeds with low or even absent activation barriers, and thus very quickly once the encounter complex has formed,^52–54^ i.e., faster than the diffusion rate constant. The available experimental effective rate coefficients applying a description in terms of bulk concentrations are thus compounded by diffusion effects.^11,55^ Note that the diffusion rate constant in 2D can be approximated as *k*_diff_ = 2*πDN*_A_/log­(*R*/σ); for diffusion-controlled reactions in 2D, the biomolecular reaction rate constant (approximately *k*_r,0_2*πσβ* ^–1^ *N*_A_) exceeds *k*_diff_, suggesting that *k*_r,0_s larger than *k*_diff_β/(2*πσN*_A_) give rise to diffusion control. Calculating this with our “baseline system” parameters, we find that the reaction is diffusion-controlled when *k*_r,0_ > 82 MHz. Therefore, the intermediate rate coefficients explored here appear clearly reasonable, while the largest ones serve the purpose of exploring the limit of instantaneous recombination in the singlet configuration.

Our model would make more concrete, biologically relevant predictions if MD-derived system parameters were included. This would allow for characteristics such as membrane flexibility and molecular shape to be included. This remains computationally expensive for the type of systematic parameter explorations undertaken here. A promising direction for future work involves extending the current model to incorporate an additional spin relaxation pathway arising from fluctuations in the recombination rate constant due to variations in the inter-radical distance. Since recombination is inherently distance-dependent, such fluctuations could contribute to relaxation dynamics, akin to the effects observed from fluctuating EED couplings. However, our current approach, based on the Nakajima–Zwanzig formalism, quickly breaks down if recombination rate fluctuations are included in the relaxation superoperator for the fast recombination rate constants relevant here. To address this, future studies will have to employ alternative simulation strategies, such as those based on the stochastic Schrödinger equation,^56,57^ which would allow for an explicit treatment of time-dependent recombination dynamics and their potential impact on MFEs in lipid radical pair systems.

## Conclusion

In this study, we investigated the viability of MFEs in lipid peroxidation through the framework of the RPM, focusing in particular on the influence of spin relaxation and EED interactions. These EED interactions, modulated by the diffusive motion of lipid peroxyl radicals, are inherently present, i.e., unavoidable, but have been largely absent or oversimplified in previous RPM-based assessments of lipid autoxidation. Here, for the first time, they are fully incorporated into a master equation treatment of spin dynamics of a pair of lipid peroxyl radicals, informed by their Brownian dynamics within a microdomain geometry, consistent with the “pickets and fences” model of biological membranes.

Our results show that even in the presence of EED couplings and Δ*g* effects, both static and fluctuating, MFEs remain feasible, provided that the average recombination rate constant, *k*_r_, is sufficiently high (on the order of hundreds of MHz) to invoke the quantum Zeno effect. This effect allows weak magnetic fields to exert pronounced influence on triplet-born radical pairs by leveraging state degeneracies and their field-induced coupling and stabilizes the spin dynamics against decoherence. Notably, F-pairs can mimic this behavior if an efficient singlet recombination pathway is available.

We identified a rich MFE landscape shaped by the interplay of competing coherent and incoherent spin dynamics: a positive low-field effect driven by the RPM and the quantum Zeno effect and high-field effects, positive or negative, depending on *k*_r_ and resulting from the antagonistic effects of the quantum Zeno-bolstered RPM and the spin relaxation mechanism. A critical *k*_r_ ∼ 700 μs^–1^ was identified for which the two opposing effects cancel each other in high fields (>10 mT) and longer correlation times, leaving only a low-field response of enhanced lipid peroxyl recombination in the mT region. This dynamic interplay provides a unifying explanation for previously conflicting experimental findings, namely reports of increased and decreased oxidative stress upon exposure to magnetic fields, and offers a predictive framework for understanding such outcomes. Because the effective recombination rate constant is highly system-dependent, shaped by factors such as diffusion coefficients, microdomain size, membrane composition, and temperature, this framework also explains the diversity of MFE responses observed in the literature. However, the scarcity of systematic experimental studies on tractable systems remains a significant limitation.

Moving forward, we stress the importance of experimental studies that span a broad and strategically selected range of magnetic field strengths, especially including the sub- to low-millitesla regime. Such measurements, when combined with theoretical modeling, would allow inference of the effective recombination regime and help clarify the biological relevance of RPM-driven MFEs. This is particularly important for assessing the potential of static magnetic field exposure in biomedical applications, including oxidative stress modulation during cryopreservation, and as a possible adjuvant in cancer therapies, where magnetic fields could augment ROS-mediated damage.

Finally, a key direction for future theoretical work lies in accounting for additional relaxation channels arising from fluctuations in the recombination rate constant itself, driven by variations in inter-radical distance, which could influence spin dynamics similarly to EED couplings. Since our current formalism, based on the Nakajima–Zwanzig approach, breaks down, future studies will require alternative methods such as stochastic Schrödinger equation simulations. Work in this direction is underway. With respect to future experimental directions, we hope that this work encourages fundamental investigations of MFEs, ideally in simple model systems such as unsaturated phospholipid vesicles, whereby the generation of ROS, lipid peroxidation decay products, oxygen consumption, or ultraweak photon emission from Russell mechanism recombination would be tangible measures. It is important that these investigations span a range of field strengths (suggested here) in order to establish a comprehensive picture, as the MFE profile is predicted to be acutely sensitive to the rate of recombination and could therefore vary from system to system. Another important area of interest is MFEs in lipid peroxidation in biomedical contexts. Here, stringent controls including sham exposures, simultaneous exposure, and sham exposure and blinded experimental protocols are recommended to avoid confounding factors and erroneous conclusions.

## Supplementary Material



## Data Availability

The data supporting the findings of this study are available in the article and its Supporting Information.

## Notes

Simulation details and additional results illustrating the variability of the magnetic field effect on the microdomain size, diffusion coefficient, and hyperfine interaction strength (PDF)
