# Supplementary material for: Quantum Zeno Effect Permits Magnetosensitivity in Lipid Peroxidation despite Fluctuating Inter-Radical Coupling
Source: JACS Au. 2026 Apr 15;6(4):2420–32. doi: 10.1021/jacsau.6c00031 (PMC13126183; doi:10.1021/jacsau.6c00031)
Supplement: Supplementary file 1 [file au6c00031_si_001.pdf]

Supporting Information to "Quantum Zeno Effect Permits Magnetosensitivity in Lipid Peroxidation Despite Fluctuating Inter-radical Coupling"

Matt C. J. Denton, Daniel R. Kattnig
Department of Physics and Living Systems Institute
University of Exeter, Stocker Road, Exeter EX4 4QD, U.K.

April 13, 2026

Suppl. Note 1: Comparing Differing System Configurations

Reminder of 'baseline system' parameters (all plots in the main article are of this particular parameter choice: $a_{\mathrm{iso}}/(2\pi) = 10.3\,\mathrm{MHz}$, $D = 0.14\,\mathrm{Å}^2\,\mathrm{ns}^{-1}$, $R = 25\,\mathrm{Å}$, $k_f = 1\,\mu\mathrm{s}^{-1}$). Justification for the range of parameters that were investigated can be found in the 'Model' section of the main manuscript, but we refer the reader to papers that justify choices of diffusion coefficient [1, 2], micro-domain radii [3], and isotropic hyperfine coupling constants [4].

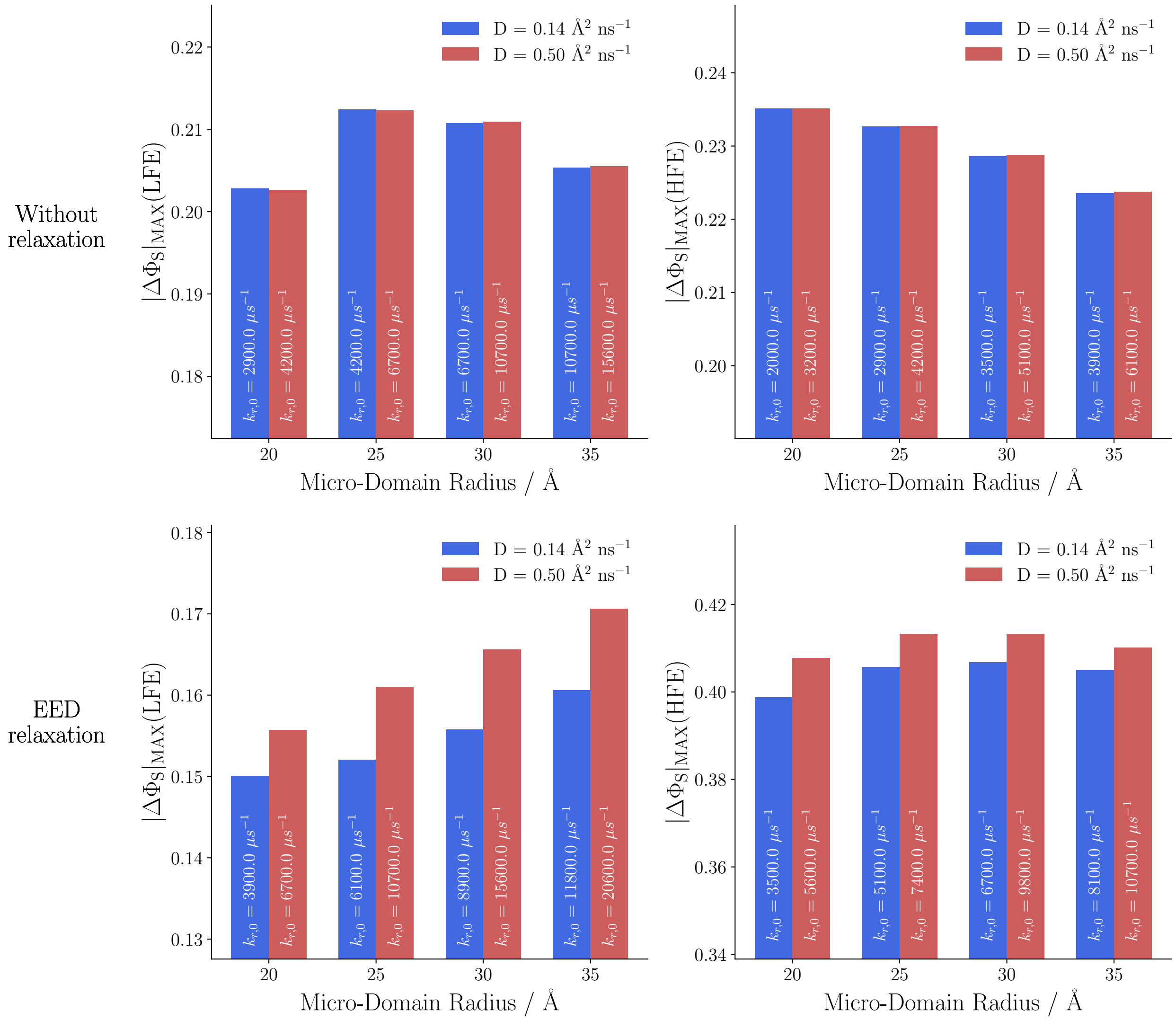

Suppl. Fig. 1: Composite figure of 4 bar plots showing the maximum singlet yield delta ($\Delta\Phi_S$) for both the low field effect (LFE) and the high field effect (HFE) for varying radii of micro-domain (R), and varying values of diffusion coefficient (D). Four different radii of the 2D lipid micro-domain, in 5Å increments from 20Å to 35Å, and two different values of diffusion coefficient, 0.14Å^{2}ns^{-1} and 0.5Å^{2}ns^{-1} were modelled, each combination of which represents an individual bar in each bar plot. The top row of plots shows results for a system where no additional relaxation channels have been incorporated into the spin dynamics, whereas the bottom row includes the relaxation effect induced by the inclusion of fluctuation in electron-electron dipolar coupling. What is clear from the bars is that the change in parameters within this range, deemed to be representative of the viable range of possible systems *in vivo*, does not cause an outsized change in the yield variability of the resulting spin dynamics. These results led to the decision being taken that, throughout the investigation, the selection of a so-called 'baseline system' set of parameters (R = 25 Å and D = 0.14 Å^{2}ns^{-1}) would result in representative outcomes which could be relied upon for analysing magnetosensitivity of this system generally. One small feature to note is that there is a degree of enhancement of both HFE and LFE in the case including EED relaxation where the micro-domain radius is increased, provided that the value of $k_{r,0}$ increases as well.

1.1. Effect of Changing Micro-Domain Radius

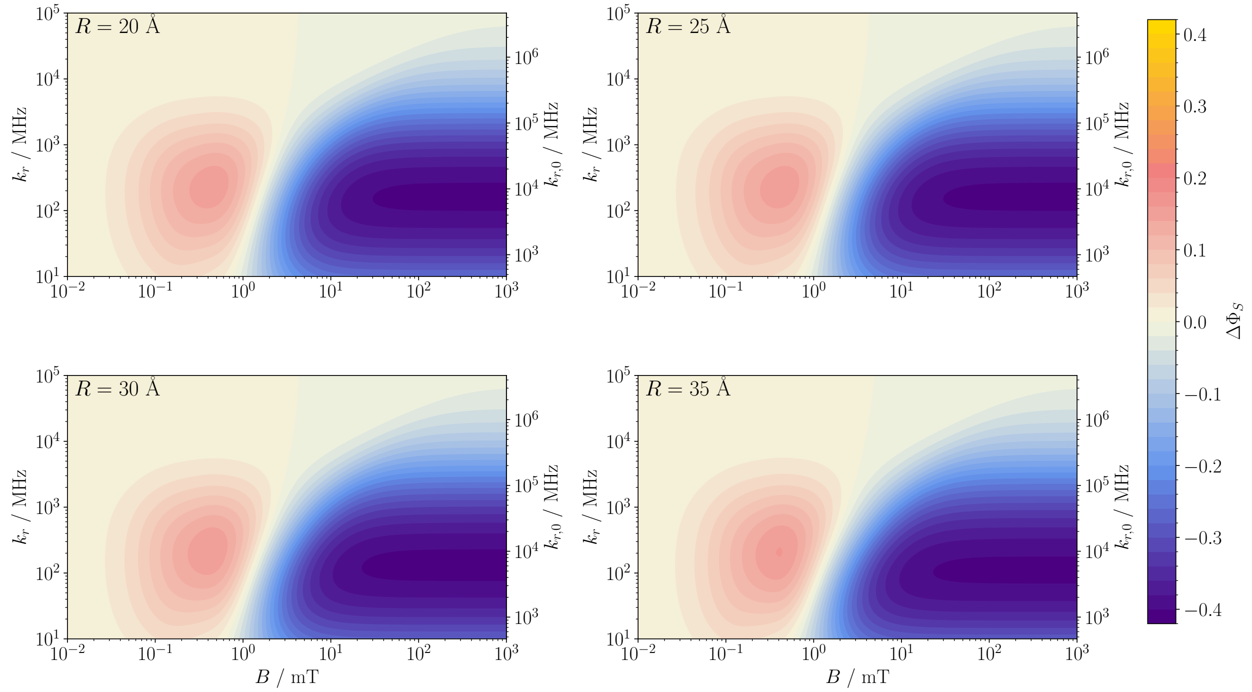

Suppl. Fig. 2: Composite figure of four heatmaps of absolute singlet yield difference $\Delta\Phi_S$ as a function of k_r and B for four different radii of the lipid micro domain in the presence of fluctuating EED coupling. The upper right figure represents the radius used in the baseline system of 25Å. There are small changes in the profile of these results, but the qualitative picture across all values of R is almost entirely unchanged. Other parameters: $a_{iso}/(2\pi) = 10.3$ MHz, $D = 0.14$ Å^{2}ns^{-1}, as in the baseline system.

1.2. Effect of Changing Hyperfine Coupling

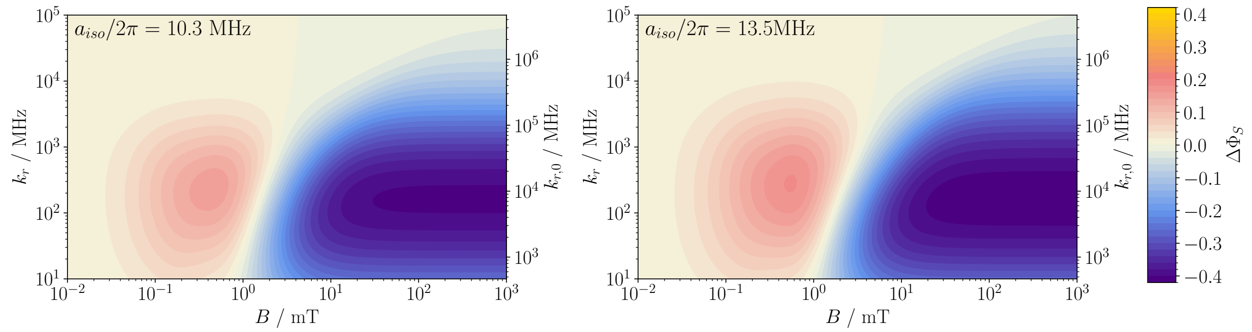

Suppl. Fig. 3: Composite figure of two heatmaps of absolute singlet yield difference $\Delta\Phi_S$ as a function of k_r and B for the two isotropic hyperfine coupling constants ($a_{iso}/(2\pi)$) of the linoleic acid peroxyl radicals, in the presence of fluctuating EED coupling. The left hand panel shows results for the 13ze isomer used in the baseline system, where $a_{iso}/(2\pi) = 10.3$ MHz. The right hand panel shows results for the 9ez isomer, where $a_{iso}/(2\pi) = 13.5$MHz. As above, there are small differences between the results for the two coupling constants, but the qualitative picture remains very consistent, hence vindicating our choosing a single isomer to display results in the main document. Other parameters: $D = 0.14$Å^2 ns^{-1}, $R = 25$ Å, as in the baseline system.

1.3. Effect of Changing Diffusion Coefficient

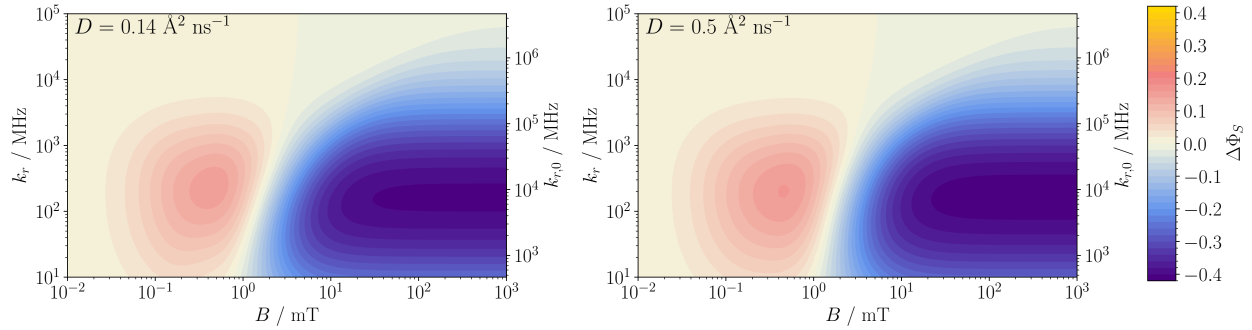

Suppl. Fig. 4: Composite figure of two heatmaps of absolute singlet yield difference $\Delta\Phi_S$ as a function of k_r and B for two values of the diffusion coefficient (D) that specifies the Brownian motion of the lipid radicals. The left hand panel shows results for the case where $D = 0.14$ Å^{2}ns^{-1}, a relatively slower rate of diffusion, and the rate used in the baseline system. The right hand panel shows results for the case where $D = 0.5$ Å^{2}ns^{-1}, a faster rate of diffusion. As in the case of varying both the micro domain radius and the isotropic hyperfine coupling constant above, there are small changes in the heatmaps, but the qualitative picture remains very consistent, hence vindicating our choosing a single value of D to display results in the main document. Other parameters: $a_{iso}/(2\pi) = 10.3$ MHz, $R = 25$ Å, as in the baseline system

1.4. Effect of Changing Radical Pair Lifetime

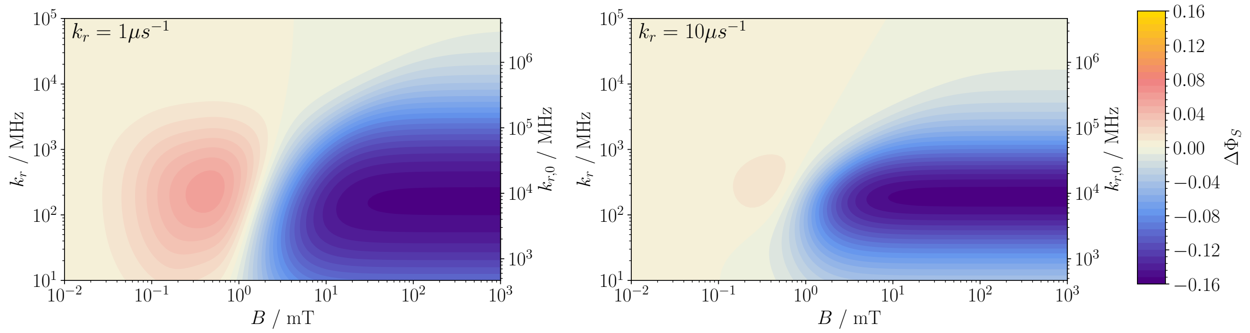

Suppl. Fig. 5: Composite figure of two heatmaps of absolute singlet yield difference $\Delta\Phi_S$ as a function of k_r and B for two values of the forward recombination rate constant k_f that inversely defines the lifetime of the radical pair. The left hand panel shows results for the case where $k_f = 1\,\mu s^{-1}$, corresponding to a 'longer lived' radical pair, and the rate used in the baseline system. The right hand panel shows results for the case where $k_f = 10\,\mu s^{-1}$, representing a 'short-lived' radical pair. As above, there are small changes in the heatmaps, most notably at lower values of magnetic field strength, where the low field effect has been reduced. However, the qualitative picture is largely the same as the baseline system, particularly at higher values of magnetic field, hence vindicating our choosing a single value of $k_f = 1\,\mu s^{-1}$ to display results in the main document. Other parameters: $a_{iso} = 10.3$ MHz, $D = 0.14\,Å^2 ns^{-1}$, $R = 25\,Å$, as in the baseline system.

Suppl. Note 2: Investigating Effects of Exchange Interaction

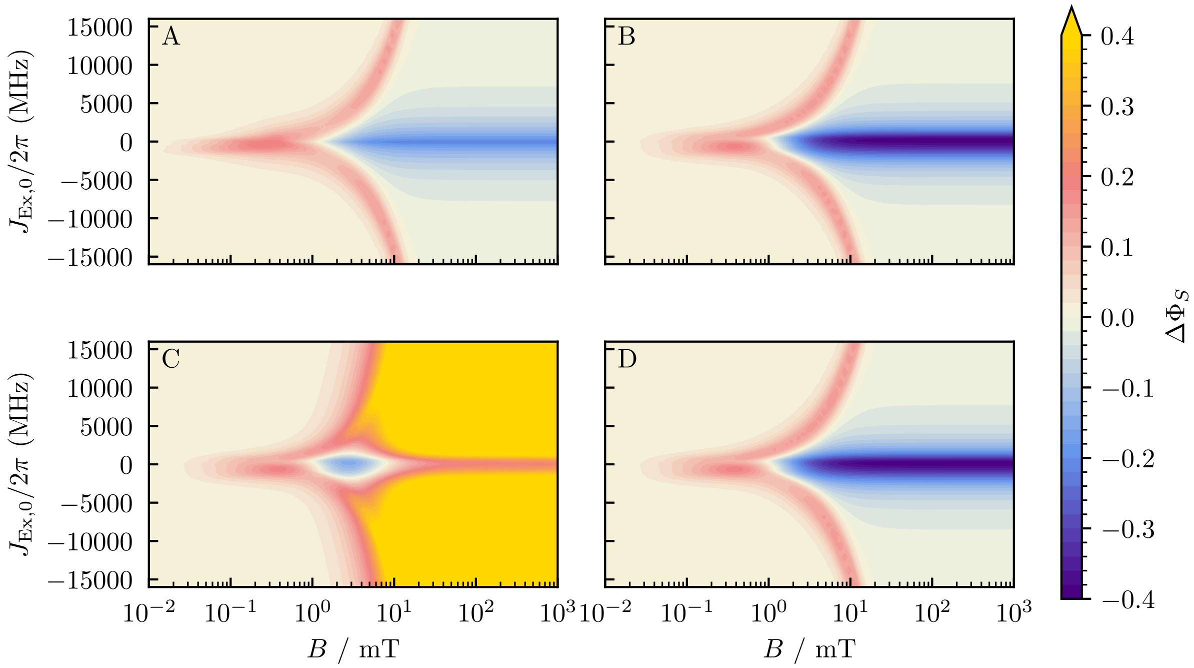

Suppl. Fig. 6: Heatmaps of maximal singlet yield difference $\Delta\Phi_{\mathrm{S}}$ as a function of $J_{\mathrm{Ex},0}/(2\pi)$ ($J_{\mathrm{Ex}} \approx 0.01 \cdot J_{\mathrm{Ex},0}$) and B under various relaxation scenarios. Panel A represents a system including HFC, EED, static exchange coupling, and Zeeman interactions. Panel B adds EED relaxation to this. Panel C additionally includes g-anisotropy and Δg-relaxation. Panel D adds fluctuating exchange coupling to the system in panel B. The colour bar maximum has been capped at 0.4, in order for subtle details not to be lost due to the high $\Delta\Phi_{\mathrm{S}}$ induced by Δg effects at high field intensities in panel C (gold regions). All panels show the characteristic branch-like structures indicative of J/D compensation described in [5]. Even with the inclusion of exchange relaxation in panel D, MFEs are resilient for lower exchange values. Other parameters: R = 35 Å, D = 0.14 Å^{2}ns^{-1}, $a_{\mathrm{iso}}/(2\pi)$ = 10.3 MHz, k_f = 1 μs^{-1}, k_r = 6000 μs^{-1}.

Suppl. Note 3: Singlet Yield Curves for an Unpolarised Initial State

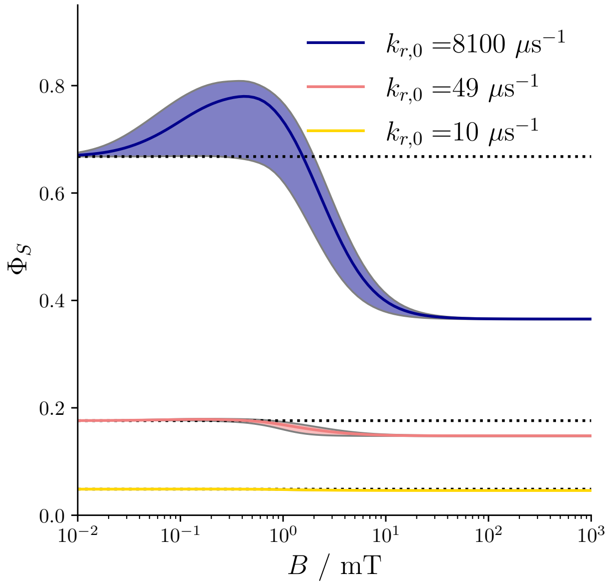

Suppl. Fig. 7: Similar to line plots shown in the main text, here we look at the singlet yield Φ_S for a lipid peroxyl RP system both in the slower rate regime and the rate range where the Zeno effect is operative. However, here the initial spin state of the RP is initialised as unpolarised, as opposed to assuming a triplet initialised configuration as can be seen in the main text. What can clearly be observed is that the same essential trends arise as in the triplet initialised case: a considerable enhancement of singlet yield is brought about by the Zeno effect, particularly in the 'low field' ranges of magnetic field.

Suppl. Note 4: Heatmaps of only Δg-Relaxation

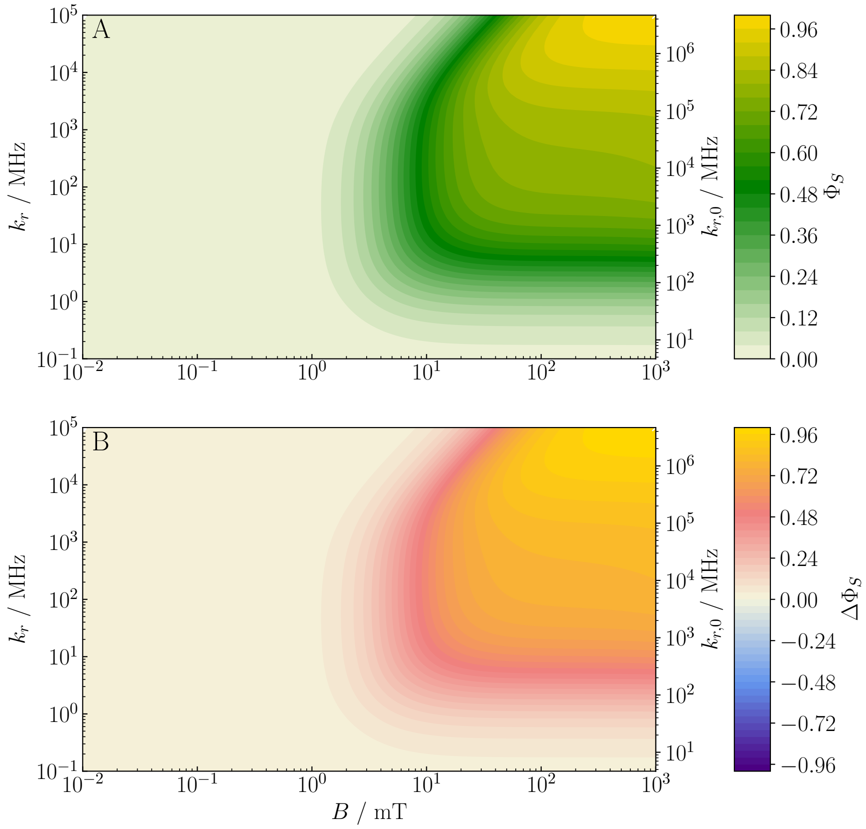

Suppl. Fig. 8: Figure showing both the change in singlet yield $\Delta\Phi_S$ from the $B = 0$ case (panel A) and absolute singlet yield Φ_S (panel B) for a system where all interactions and relaxation pathways other than those induced by g-tensor anisotropy and Δg-relaxation have been removed. Two different colour schemes have been used in order to reflect the difference in the quantity being reported in each respective panel. Clearly, on the right hand side of each plot, the higher values of B drive the dominant effect brought about by Δg-relaxation to drastically increase singlet yield. This clearly shows that the same feature that appears in the upper right corner in Fig. 6 is indeed brought about by these effects, and not EED relaxation or any other mechanism included in the system investigated there. Hence, particularly in 'high field' cases, it is imperative for investigations of these systems to include g-tensor anisotropy and Δg-relaxation.

Suppl. Note 5: Varying Δg Correlation Time

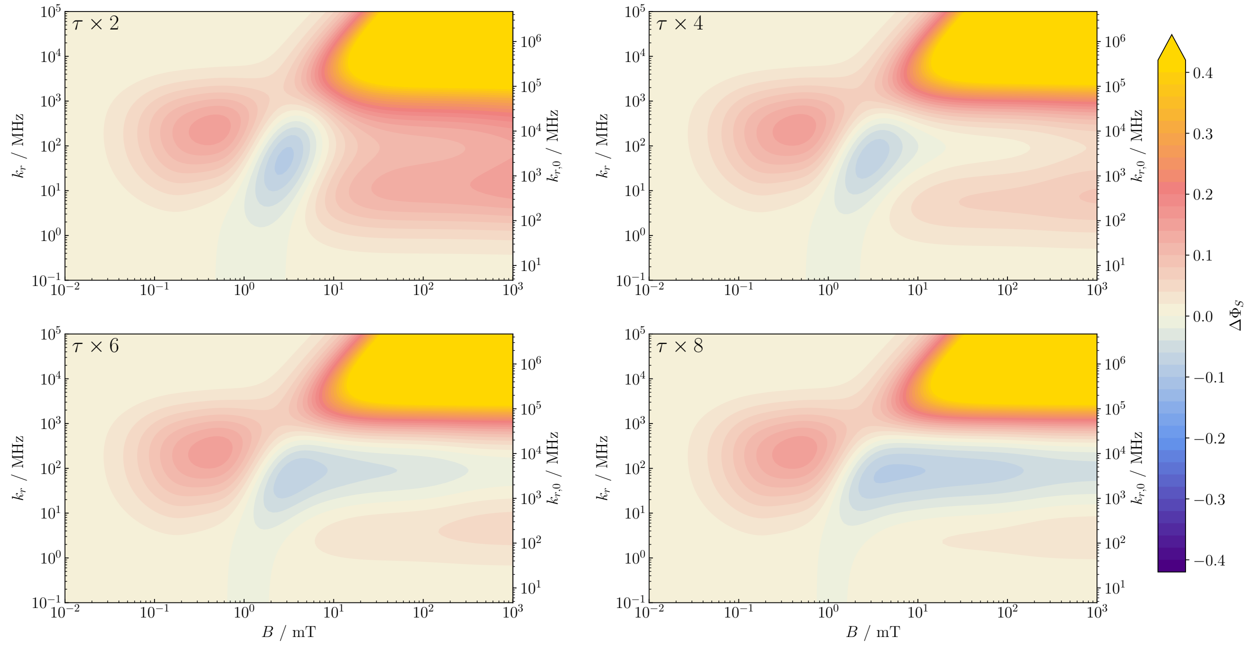

Suppl. Fig. 9: Heatmap plots showing associated MFE, $\Delta\Phi_S$, as a function of the singlet recombination rate constant, expressed as k_r (left axis) and $k_{r,0}$ (right axis), and the magnetic field strength, B, for the 'baseline system' with g-anisotropy and Δg-relaxation included on top of EED-induced spin relaxation. These plots show the results for increasing values of τ for the Δg-relaxation (multiplier indicated in the top corner of each subplot). Once τ is quadrupled, there emerges a region of net 0 MFE that extends into the high field regime, aligning with experimental results showing MFEs at low field values without MFEs at high field values. The results are in line with a model employing slightly longer correlation times than used in [6], increased to a degree that is plausibly associated with a more complex membrane environment.

Suppl. Note 6: Benchmarking Nakajima-Zwanzig Equations

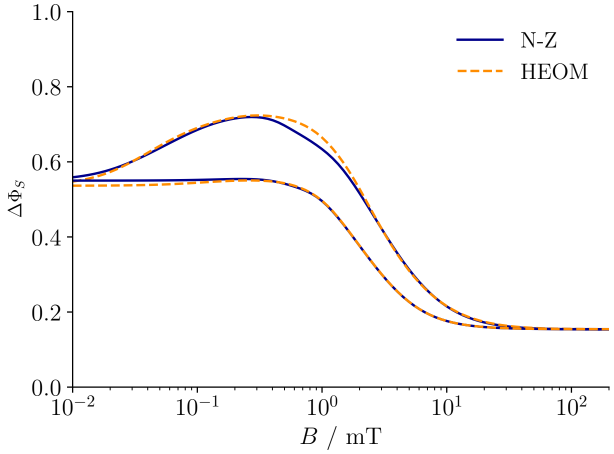

Suppl. Fig. 10: Plot of recombination yield Φ_S as a function of field strength B for a lipid peroxyl radical pair with the following system parameters: $a_{iso}/(2\pi) = 10.3\,\text{MHz}$; $D = 0.14\,\text{Å}^2\text{ns}^{-1}$; $R = 20\,\text{Å}$; $k_{r,0} = 5591\,\mu\text{s}^{-1}$; $k_f = 1\,\mu\text{s}^{-1}$. Solid lines show results obtained using the NZ approach, whereas dotted lines show results obtained using the hierarchical equations of motion (HEOM) [7]. Data series for field oriented parallel (top lines) and perpendicular (bottom lines) to the membrane plane are plotted here. This shows that there is good agreement between the two approaches, validating the use of our NZ-derived approach to evaluate MFEs in these systems, at a fraction of the computational cost of HEOM-derived methods. This is what made a broad sweep of varied system parameters across ranges of k_r and B values possible. Calculations used a hierarchy width of 3 and truncation level of 5, thus including 56 Auxiliary Density Operators (ADOs) and giving rise to a total problem dimension of 14,336 (vs. 256 for the NZ approach). Convergence was verified by increasing the truncation level to 20 (1771 ADOs; total dimension 453,376). All dipolar coupling fluctuations were represented as Ornstein-Uhlenbeck processes with a common correlation time of 100 ns. The dipolar coupling covariances were: $\langle \Delta D_{xx}^2 \rangle/(4\pi^2) = 26451\,\text{MHz}^2$; $\langle \Delta D_{xy}^2 \rangle/(4\pi^2) = 24159\,\text{MHz}^2$; $\langle \Delta D_{xx} \Delta D_{yy} \rangle/(4\pi^2) = -21796\,\text{MHz}^2$; $\langle \Delta D_{xx} \Delta D_{zz} \rangle/(4\pi^2) = -4655\,\text{MHz}^2$; $\langle \Delta D_{zz}^2 \rangle/(4\pi^2) = 9311\,\text{MHz}^2$. Mean values were $\langle D_{xx} \rangle/(2\pi) = -8.80\,\text{MHz}$ and $\langle D_{zz} \rangle/(2\pi) = 17.6\,\text{MHz}$.

Suppl. Note 7: Table of System Parameters

Parameters of the form D_{ij} denote elements of the dipolar coupling tensor, and $f(r) = \exp(-\beta(r_{1,2} - \sigma))$.

D_{xx}/MHz	D_{xy}/MHz	D_{yy}/MHz	D_{zz}/MHz	$f(r)$/MHz
-6.02	0.00277	-5.92	11.9	0.0214

Covariance Function	Covariance	τ_{eff}
$\langle (D_{xx} - \bar{D}_{xx})^2 \rangle$ or $\langle (D_{yy} - \bar{D}_{yy})^2 \rangle$	417 MHz^2	0.132 μs
$\langle (D_{xy} - \bar{D}_{xy})^2 \rangle$	370 MHz^2	0.128 μs
$\langle (D_{xx} - \bar{D}_{xx})(D_{yy} - \bar{D}_{yy}) \rangle$ or $\langle (D_{yy} - \bar{D}_{yy})(D_{xx} - \bar{D}_{xx}) \rangle$	-324 MHz^2	0.124 μs
$\langle (D_{xx} - \bar{D}_{xx})(D_{zz} - \bar{D}_{zz}) \rangle$ or $\langle (D_{yy} - \bar{D}_{yy})(D_{zz} - \bar{D}_{zz}) \rangle$ or $\langle (D_{zz} - \bar{D}_{zz})(D_{xx} - \bar{D}_{xx}) \rangle$ or $\langle (D_{zz} - \bar{D}_{zz})(D_{yy} - \bar{D}_{yy}) \rangle$	-93.5 MHz^2	0.160 μs
$\langle (D_{zz} - \bar{D}_{zz})^2 \rangle$	187 MHz^2	0.160 μs
$\langle (f(r) - \bar{f}(r))^2 \rangle$	0.0106	0.0295 μs
$\langle (f(r) - \bar{f}(r))(D_{zz} - \bar{D}_{zz}) \rangle$	0.966 MHz	0.0933 μs

Table 1: Coefficients of the multi-exponential fitting of the correlation functions.

τ_i (μs)	$c_i^{D_{xx}D_{xx}}$	$c_i^{D_{xy}D_{xy}}$	$c_i^{D_{xx}D_{yy}}$	$c_i^{D_{xx}D_{zz}}$	$c_i^{D_{zz}D_{zz}}$	$c_i^{f(r)f(r)}$	$c_i^{f(r)D_{zz}}$
$1.00 \cdot 10^{-4}$	$2.35 \cdot 10^{-14}$	$1.83 \cdot 10^{-2}$	$-1.97 \cdot 10^{-13}$	$-5.40 \cdot 10^{-2}$	$1.08 \cdot 10^{-1}$	$1.25 \cdot 10^{-5}$	$2.30 \cdot 10^{-8}$
$2.46 \cdot 10^{-4}$	$2.65 \cdot 10^{-1}$	$6.86 \cdot 10^{-8}$	$-5.42 \cdot 10^{-14}$	$-1.05 \cdot 10^{-13}$	$7.20 \cdot 10^{-14}$	$5.53 \cdot 10^{-4}$	$8.54 \cdot 10^{-3}$
$6.05 \cdot 10^{-4}$	$6.59 \cdot 10^{-1}$	1.21	-1.23	$-6.94 \cdot 10^{-19}$	$4.68 \cdot 10^{-19}$	$1.91 \cdot 10^{-3}$	$3.26 \cdot 10^{-2}$
$1.49 \cdot 10^{-3}$	6.30	3.85	-2.15	-3.43	6.86	$2.69 \cdot 10^{-3}$	$1.10 \cdot 10^{-1}$
$3.66 \cdot 10^{-3}$	$1.49 \cdot 10^{1}$	$1.52 \cdot 10^{1}$	$-1.46 \cdot 10^{1}$	$-9.95 \cdot 10^{-1}$	1.99	$2.05 \cdot 10^{-3}$	$1.18 \cdot 10^{-1}$
$9.02 \cdot 10^{-3}$	$5.21 \cdot 10^{1}$	$4.21 \cdot 10^{1}$	$-3.23 \cdot 10^{1}$	$-1.93 \cdot 10^{1}$	$3.87 \cdot 10^{1}$	$1.63 \cdot 10^{-3}$	$2.28 \cdot 10^{-1}$
$2.22 \cdot 10^{-2}$	$1.31 \cdot 10^{2}$	$1.24 \cdot 10^{2}$	$-1.20 \cdot 10^{2}$	$-1.10 \cdot 10^{1}$	$2.21 \cdot 10^{1}$	$7.77 \cdot 10^{-4}$	$1.40 \cdot 10^{-1}$
$5.46 \cdot 10^{-2}$	$1.59 \cdot 10^{2}$	$1.41 \cdot 10^{2}$	$-1.19 \cdot 10^{2}$	$-4.05 \cdot 10^{1}$	$8.10 \cdot 10^{1}$	$7.03 \cdot 10^{-4}$	$2.32 \cdot 10^{-1}$
$1.34 \cdot 10^{-1}$	$5.27 \cdot 10^{1}$	$4.25 \cdot 10^{1}$	$-3.45 \cdot 10^{1}$	$-1.82 \cdot 10^{1}$	$3.64 \cdot 10^{1}$	$2.50 \cdot 10^{-4}$	$9.71 \cdot 10^{-2}$
$3.30 \cdot 10^{-1}$	~ 0	~ 0	~ 0	~ 0	~ 0	$2.33 \cdot 10^{-7}$	~ 0
$8.13 \cdot 10^{-1}$	~ 0	~ 0	~ 0	~ 0	~ 0	$2.72 \cdot 10^{-7}$	~ 0
2.00	~ 0	~ 0	~ 0	~ 0	~ 0	$1.75 \cdot 10^{-7}$	~ 0

7.1. Δg-Relaxation Parameters

The following parameters were produced through DFT and MD analysis in an investigation by Grüning et al. [8].

$$\mathbf{g} = \begin{bmatrix} 2.0062 & 2.6065 \cdot 10^{-3} & -4.3308 \cdot 10^{-4} \\ 2.6065 \cdot 10^{-3} & 2.0054 & 0 \\ -4.3308 \cdot 10^{-4} & 0 & 2.0274 \end{bmatrix}$$

$$\mathrm{Cov}(g) = \begin{bmatrix} 4.26\cdot10^{-3} & -5.72\cdot10^{-5} & -3.53\cdot10^{-6} & -5.72\cdot10^{-5} & -1.80\cdot10^{-3} & -8.10\cdot10^{-5} & -3.53\cdot10^{-6} & -8.10\cdot10^{-5} & -2.46\cdot10^{-3} \\ -5.72\cdot10^{-5} & 3.02\cdot10^{-3} & 1.45\cdot10^{-5} & 3.02\cdot10^{-3} & 1.25\cdot10^{-5} & -5.59\cdot10^{-5} & 1.45\cdot10^{-5} & -5.59\cdot10^{-5} & 4.46\cdot10^{-5} \\ -3.53\cdot10^{-6} & 1.45\cdot10^{-5} & 3.85\cdot10^{-3} & 1.45\cdot10^{-5} & -3.17\cdot10^{-5} & -3.42\cdot10^{-5} & 3.85\cdot10^{-3} & -3.42\cdot10^{-5} & 3.53\cdot10^{-5} \\ -5.72\cdot10^{-5} & 3.02\cdot10^{-3} & 1.45\cdot10^{-5} & 3.02\cdot10^{-3} & 1.25\cdot10^{-5} & -5.59\cdot10^{-5} & 1.45\cdot10^{-5} & -5.59\cdot10^{-5} & 4.46\cdot10^{-5} \\ -1.80\cdot10^{-3} & 1.25\cdot10^{-5} & -3.17\cdot10^{-5} & 1.25\cdot10^{-5} & 4.34\cdot10^{-3} & 5.07\cdot10^{-5} & -3.17\cdot10^{-5} & 5.07\cdot10^{-5} & -2.54\cdot10^{-3} \\ -8.10\cdot10^{-5} & -5.59\cdot10^{-5} & -3.42\cdot10^{-5} & -5.59\cdot10^{-5} & 5.07\cdot10^{-5} & 3.75\cdot10^{-3} & -3.42\cdot10^{-5} & 3.75\cdot10^{-3} & 3.03\cdot10^{-5} \\ -3.53\cdot10^{-6} & 1.45\cdot10^{-5} & 3.85\cdot10^{-3} & 1.45\cdot10^{-5} & -3.17\cdot10^{-5} & -3.42\cdot10^{-5} & 3.85\cdot10^{-3} & -3.42\cdot10^{-5} & 3.53\cdot10^{-5} \\ -8.10\cdot10^{-5} & -5.59\cdot10^{-5} & -3.42\cdot10^{-5} & -5.59\cdot10^{-5} & 5.07\cdot10^{-5} & 3.75\cdot10^{-3} & -3.42\cdot10^{-5} & 3.75\cdot10^{-3} & 3.03\cdot10^{-5} \\ -2.46\cdot10^{-3} & 4.46\cdot10^{-5} & 3.53\cdot10^{-5} & 4.46\cdot10^{-5} & -2.54\cdot10^{-3} & 3.03\cdot10^{-5} & 3.53\cdot10^{-5} & 3.03\cdot10^{-5} & 4.99\cdot10^{-3} \end{bmatrix}$$

Table 2: Effective correlation times for the g-tensor of the average peroxide lipid. Permutations of indices share the same value.

Component	τ (ns)
$\tau(g_{xx,xx})$	$6.71\cdot10^{-1}$
$\tau(g_{xx,xy})$	$1.12\cdot10^{-1}$
$\tau(g_{xx,xz})$	$2.17\cdot10^{-2}$
$\tau(g_{xx,yy})$	$6.20\cdot10^{-1}$
$\tau(g_{xx,yz})$	$2.34\cdot10^{-2}$
$\tau(g_{xx,zz})$	$2.90\cdot10^{-1}$
$\tau(g_{xy,xy})$	$8.24\cdot10^{-1}$
$\tau(g_{xy,xz})$	$1.34\cdot10^{0}$
$\tau(g_{xy,yy})$	$8.49\cdot10^{-1}$
$\tau(g_{xy,yz})$	2.01
$\tau(g_{xy,zz})$	$4.12\cdot10^{-2}$
$\tau(g_{xz,xz})$	1.90
$\tau(g_{xz,yy})$	1.43
$\tau(g_{xz,yz})$	$2.95\cdot10^{-1}$
$\tau(g_{xz,zz})$	$5.43\cdot10^{-2}$
$\tau(g_{yy,yy})$	$9.77\cdot10^{-1}$
$\tau(g_{yy,yz})$	$3.86\cdot10^{-3}$
$\tau(g_{yy,zz})$	$6.74\cdot10^{-1}$
$\tau(g_{yz,yz})$	$6.15\cdot10^{-1}$
$\tau(g_{yz,zz})$	1.15
$\tau(g_{zz,zz})$	$6.38\cdot10^{-1}$

7.2. Coherence Times

Table 3: Coherence times of $S_{1,x}$ and $S_{1,z}$ for all permutations of micro-domain radius R and diffusion coefficient D. These times are associated with electron-electron dipolar coupling induced relaxation.

D (Å^{2}ns^{-1})	R (Å)	T($S_{1,x}$) (ns)	T($S_{1,z}$) (ns)
0.14	20	3220	218
0.14	25	5760	218
0.14	30	8970	218
0.14	35	12800	213
0.5	20	1410	146
0.5	25	2370	153
0.5	30	3560	162
0.5	35	4990	172

Table 4: Coherence times of $S_{1,x}$ and $S_{1,z}$ for several values of applied magnetic field B, when including the effects of Δg-relaxation. These times do not vary when changing system parameters other than B.

B (mT)	T($S_{1,x}$) (ns)	T($S_{1,z}$) (ns)
1	23800	14700
10	350	790
100	5.56	508
1000	0.0562	505

References

1. Jacobson, K., Ishihara, A. & Inman, R. Lateral Diffusion of Proteins in Membranes. *Annual Review of Physiology* **49,** 163–175. ISSN: 1545-1585 (Mar. 1987).
2. Jan Akhunzada, M. *et al.* Interplay between lipid lateral diffusion, dye concentration and membrane permeability unveiled by a combined spectroscopic and computational study of a model lipid bilayer. *Scientific Reports* **9.** ISSN: 2045-2322 (Feb. 2019).
3. Kusumi, A. *et al.* Paradigm shift of the plasma membrane concept from the two-dimensional continuum fluid to the partitioned fluid: high-speed single-molecule tracking of membrane molecules. *Annu. Rev. Biophys. Biomol. Struct.* **34,** 351–378 (2005).
4. Sampson, C., Keens, R. H. & Kattnig, D. R. On the magnetosensitivity of lipid peroxidation: Two-versus three-radical dynamics. *Physical Chemistry Chemical Physics* **21,** 13526–13538 (2019).
5. Efimova, O. & Hore, P. Role of exchange and dipolar interactions in the radical pair model of the avian magnetic compass. *Biophysical journal* **94,** 1565–1574 (2008).
6. Kabuto, H., Yokoi, I., Ogawa, N., Mori, A. & Liburdy, R. P. Effects of magnetic fields on the accumulation of thiobarbituric acid reactive substances induced by iron salt and H2O2 in mouse brain homogenates or phosphotidylcholine. *Pathophysiology* **7,** 283–288 (2001).
7. Tanimura, Y. Numerically "exact" approach to open quantum dynamics: The hierarchical equations of motion (HEOM). *The Journal of Chemical Physics* **153.** ISSN: 1089-7690 (July 2020).
8. Grüning, G., Gerhards, L., Sampson, C., Kattnig, D. R. & Solov'yov, I. A. Spin Relaxation Does Not Preclude Magnetic Field Effects on Lipid Autoxidation. *ACS Central Science* **12,** 49–62. ISSN: 2374-7951 (Dec. 2025).
